# Integrated Epidemiological and Extracellular Vesicle Profiling Identifies miR-126-3p, miR-21-5p, miR-92a-3p and SNAIL as Candidate Biomarkers of Endothelial Dysfunction and Cardio-Metabolic Risk

**DOI:** 10.3390/ijms27156966

**Published:** 2026-08-03

**Authors:** Luis Alberto Gómez-Grosso, Gladis Estella Montoya Ortiz, Jhon Jairo Osorio-Méndez, María Paula Aulestia-Vacca, Shirley Natali Iza Rodríguez, Ana Yiby Forero Torres

**Affiliations:** 1 Molecular Physiology Group, Sub-Direction of Scientific and Technological Research, Direction of Public Health Research, National Institute of Health, Bogotá 111321, Colombia; gmontoya@ins.gov.co (G.E.M.O.); josoriom@ins.gov.co (J.J.O.-M.); maulestia@ins.gov.co (M.P.A.-V.); 2 Department of Physiological Sciences, Faculty of Medicine, Universidad Nacional de Colombia, Bogotá 111321, Colombia; 3 Master in Biochemistry Program, Department of Physiological Sciences, Medicine Faculty, Universidad Nacional de Colombia, Bogotá 111321, Colombia; siza@unal.edu.co; 4 Nutrition Group, Sub-Direction of Scientific and Technological Research, Direction of Public Health Research, National Institute of Health, Bogotá 111321, Colombia; aforero@ins.gov.co

**Keywords:** extracellular vesicles, miRNAs, cardiometabolic diseases, biomarkers, snail, type 2 diabetes, endothelial dysfunction, vulnerable populations

## Abstract

Non-communicable diseases (NCDs), including type 2 diabetes mellitus (DM), hypertension (HTA), obesity (OB), and cardiovascular disease (CVD), represent a major global health burden and disproportionately affect socially vulnerable populations. Small extracellular vesicles (EVs) are stable carriers of proteins and regulatory RNAs that may reflect endothelial dysfunction and cardiometabolic stress. This study explored plasma-derived EV-associated biomarkers in adults from La Guajira, Colombia, with emphasis on miR-126-3p, miR-21-5p, miR-92a-3p, and SNAIL. Anthropometric, clinical, and biochemical data were obtained from a cross-sectional cohort. Representative plasma samples were selected for EVs isolation by size-exclusion chromatography. Vesicles were characterized by nanoparticle tracking analysis, scanning transmission electron microscopy, protein quantification, Western blotting, and bead-based flow cytometry. Candidate miRNAs were identified by small RNA sequencing and validated by stem-loop RT-qPCR. Bioinformatic enrichment analyses were performed using miRNet 2.0 and KEGG pathway analysis. The epidemiological cohort showed a high cardiometabolic burden, with waist-to-height ratio (WHtR) and triglyceride-glucose (TyG) index emerging as key markers of central adiposity and metabolic dysfunction. Plasma-derived EVs displayed the expected nanoscale morphology and expressed canonical vesicle markers. Protein cargo analyses revealed EMT- and remodeling-associated proteins, including SNAIL and GAL-3. EV-associated miR-126-3p, miR-21-5p, and miR-92a-3p were enriched predominantly in hypertensive and hypertensive-diabetic groups. In an exploratory sub-cohort, CD31-positive EVs showed a trend toward increased expression in individuals with previous myocardial infarction or stroke (*p* = 0.057). KEGG enrichment analysis linked the identified miRNA signature to pathways involved in endothelial dysfunction, inflammation, vascular remodeling, apoptosis, and extracellular matrix organization. Plasma-derived EVs from individuals with cardiometabolic disorders carry molecular signatures consistent with endothelial stress and vascular remodeling. These findings identify miR-126-3p, miR-21-5p, miR-92a-3p, SNAIL, and CD31-positive EVs as exploratory candidate biomarkers that require validation in larger prospective cohorts.

## 1. Introduction

Non-communicable diseases (NCDs), particularly cardiovascular disease (CVD), type 2 diabetes mellitus (DM), obesity (OB), and hypertension (HTA), remain the leading causes of morbidity and mortality worldwide, accounting for nearly three-quarters of all global deaths [1,2,3,4,5]. The burden of these disorders is especially pronounced in low- and middle-income countries, where socioeconomic vulnerability, limited healthcare access, food insecurity, and environmental stressors contribute to accelerated disease progression and poor clinical outcomes [1,3,4].

Cardiometabolic disorders are increasingly recognized as interconnected manifestations of a common pathophysiological continuum characterized by insulin resistance, visceral adiposity, endothelial dysfunction, chronic low-grade inflammation, and vascular remodeling. Endothelial dysfunction represents one of the earliest detectable events in this process and precedes the development of overt atherosclerosis, myocardial infarction, stroke, chronic kidney disease, and heart failure [6,7,8].

Small extracellular vesicles (EVs) have emerged as critical mediators of intercellular communication and promising liquid-biopsy platforms for cardiovascular and metabolic diseases. These nanosized membrane vesicles transport proteins, lipids, messenger RNAs, and microRNAs (miRNAs), reflecting the physiological and pathological state of their cells of origin [9,10,11,12,13]. Owing to their remarkable stability in biological fluids and their ability to protect molecular cargo from degradation, EVs constitute attractive candidates for biomarker discovery and disease monitoring [14,15,16].

Among EV-associated miRNAs, miR-126-3p is of particular interest because of its central role in endothelial homeostasis, angiogenesis, vascular repair, and maintenance of endothelial integrity [17,18,19,20,21]. Alterations in circulating miR-126 levels have been associated with endothelial dysfunction, atherosclerosis, hypertension, diabetes mellitus, and increased cardiovascular risk. Similarly, EVs carrying the endothelial-associated marker CD31 (PECAM-1) participate in leukocyte transmigration, mechano-transduction, angiogenesis, and vascular integrity and have been proposed as indicators of endothelial injury and residual cardiovascular risk [22,23].

Another emerging pathway involved in cardiometabolic disease progression is endothelial-to-mesenchymal transition (EndMT), a process closely related to epithelial-to-mesenchymal transition (EMT). The transcription factor SNAIL is a master regulator of EMT/EndMT and contributes to fibrosis, vascular remodeling, inflammation, and atherosclerotic progression [22,24,25]. Although SNAIL has been extensively investigated in cancer biology, its presence within circulating plasma-derived extracellular vesicles remains poorly characterized [26,27].

Therefore, the present study aimed to identify and characterize plasma-derived EV-associated biomarkers linked to endothelial dysfunction and cardiometabolic disease. By integrating vesicle characterization, miRNA profiling, surface marker analyses, and bioinformatic approaches, we evaluated the potential relevance of miR-126-3p, miR-21-5p, miR-92a-3p, CD31, and SNAIL as candidate biomarkers associated with cardiovascular and cardiometabolic risk in a vulnerable population from La Guajira, Colombia.

## 2. Results

### 2.1. Study Population and Characterization of Plasma-Derived Small Extracellular Vesicles

The study included 602 adults from Riohacha, Maicao, and Manaure (La Guajira, Colombia), with a mean age of 58.6 ± 13.1 years; 65% were women. Participants were classified into healthy controls (Ctrl), obesity (OB), hypertension (HTA), type 2 diabetes mellitus (DM), combined hypertension and diabetes (HTA+DM), previous myocardial infarction (AMI), and ischemic stroke groups.

As summarized in Table 1, the cohort exhibited a high prevalence of cardiometabolic risk factors, including central adiposity, hypertriglyceridemia, impaired glucose metabolism, and elevated homocysteine levels. Mean Waist-to-Height Ratio (WHtR) was positively associated with triglycerides, fasting glucose, hypertension, and the Triglyceride—Glucose (TyG) index, while multivariable analysis identified TyG as the possible independent predictor of elevated WHtR.

The multivariable logistic regression analysis revealed a strong and independent association between systemic insulin resistance and central adiposity. When adjusting for potential confounding variables, an elevated TyG index was identified as a significant independent predictor of an increased WHtR (rho = 0.0968; *p* = 0.0177).

### 2.2. Isolation, Quantification, and Characterization of Plasma-Derived Small Extracellular Vesicles

**Figure 1:**
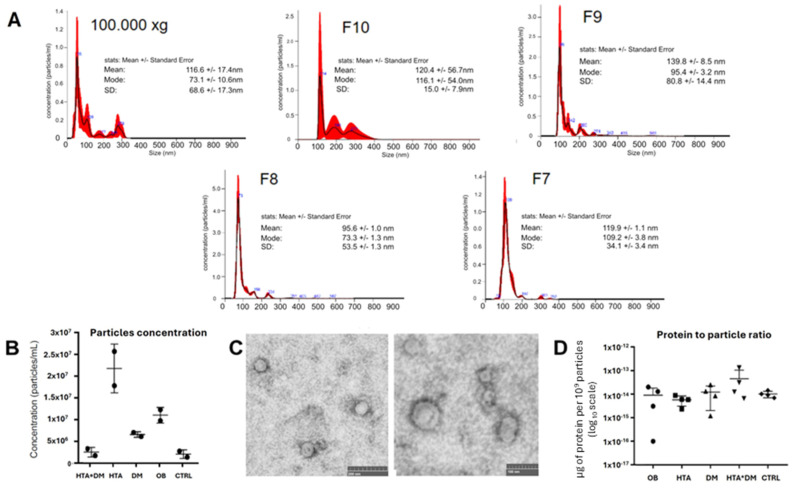
Isolation, quantification, and characterization of extracellular vesicles derived from blood plasmas. (**A**) Representative images of the NTA characterization of EVs obtained by ultracentrifugation (UC-NTC image as a methodological comparison with SEC EVs images) and size-exclusion chromatography (SEC) from patient samples. (**B**) Particle concentration in representative patient samples. (**C**) Representative STEM images of EVs. Scale bar: 100 nm. (**D**) Protein concentration to particle ratio in representative patient samples. Inferior panel: The ratio of particle concentration to protein concentration (10^9^ particles/µg protein) was calculated for each sample (*n* = 4 per group). Particle concentrations were obtained from group-level NTA measurements, whereas protein concentrations were measured individually. Values are shown for descriptive characterization of extracellular vesicle preparations. This parameter was included as an indicator of vesicle preparation enrichment and quality.

Plasma-derived extracellular vesicles (EVs) were isolated by size-exclusion chromatography (SEC) (Figure 1). To validate our isolation approach, the size distribution profiles of the size-exclusion chromatography (SEC) fractions (F7 to F10) were compared via Nanoparticle Tracking Analysis (NTA) against a reference control obtained by conventional differential ultracentrifugation (UC) (Figure 1A—indicated as 100,000 xg). Nanoparticle tracking analysis confirmed the recovery of heterogeneous nanosized particles within the expected size range for EVs-enriched preparations. Representative samples showed mean diameters of 139.8 nm and 95.6 nm, with modal diameters of 116.1 nm and 73.3 nm, respectively.

Scanning transmission electron microscopy confirmed the presence of rounded membrane-delimited vesicular structures predominantly below 200 nm (Figure 1C), supporting the successful enrichment and structural preservation of plasma-derived EVs. Particle concentration varied among clinical groups, with the highest values observed in samples from hypertensive participants.

Total protein results revealed variable protein concentrations across all cardio- metabolic conditions compared with the control group, which exhibited a mean protein concentration of 70.25 µg/mL. Protein levels varied across cardiometabolic groups, with the highest mean concentration observed in hypertension (153.55 µg/mL), followed by HTA+DM (117.71 µg/mL), obesity (110.90 µg/mL), DM (88.83 µg/mL), and controls (70.25 µg/mL) (Figure 1D). Although these differences did not reach statistical significance, the trend suggested qualitative differences in vesicular and co-isolated plasma cargo among cardiometabolic conditions.

**Figure 2:**
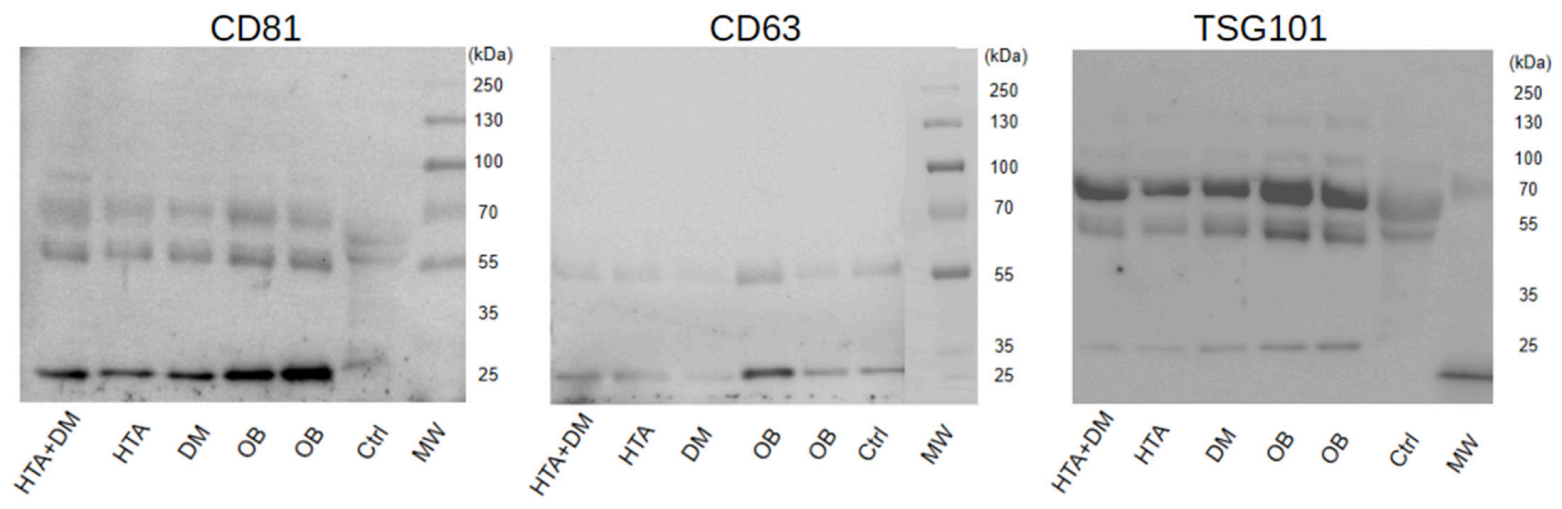
Western blot characterization of plasma-derived small extracellular vesicles (EVs) showing expression of CD81, CD63 and TSG101. Western blot (WB) analysis of the extracellular vesicle markers CD81, CD63 and TSG101 in plasma-derived EVs isolated from study participants. Lanes 1–6 represent individual plasma samples. The duplicate ‘OB’ lanes represent samples from two distinct individuals within the obesity group, included to demonstrate the biological variability and differential signaling. Markers were consistently detected across samples, confirming the enrichment and vesicular identity of the isolated EVs. Variations in band intensity were observed among participants, while higher-molecular-weight bands and multiprotein complexes were also detected, suggesting the presence of biologically active vesicular protein assemblies. The presented blots are representative of three independent vesicles’ isolation.

Western blot analysis confirmed the presence of canonical EV markers, including CD81, CD63 and TSG101 across plasma-derived preparations (Figure 2). Variability in band intensity was observed among samples, whereas higher-molecular-weight immunoreactive bands were also detected, suggesting the presence of vesicle-associated protein complexes or co-migrating molecular assemblies.

Because EVs can transport signaling proteins and regulatory molecules between cells, we next evaluated whether plasma-derived EVs contained proteins associated with epithelial-to-mesenchymal transition (EMT) and endothelial-to-mesenchymal transition (EndMT), processes implicated in vascular remodeling and fibrosis.

**Figure 3:**
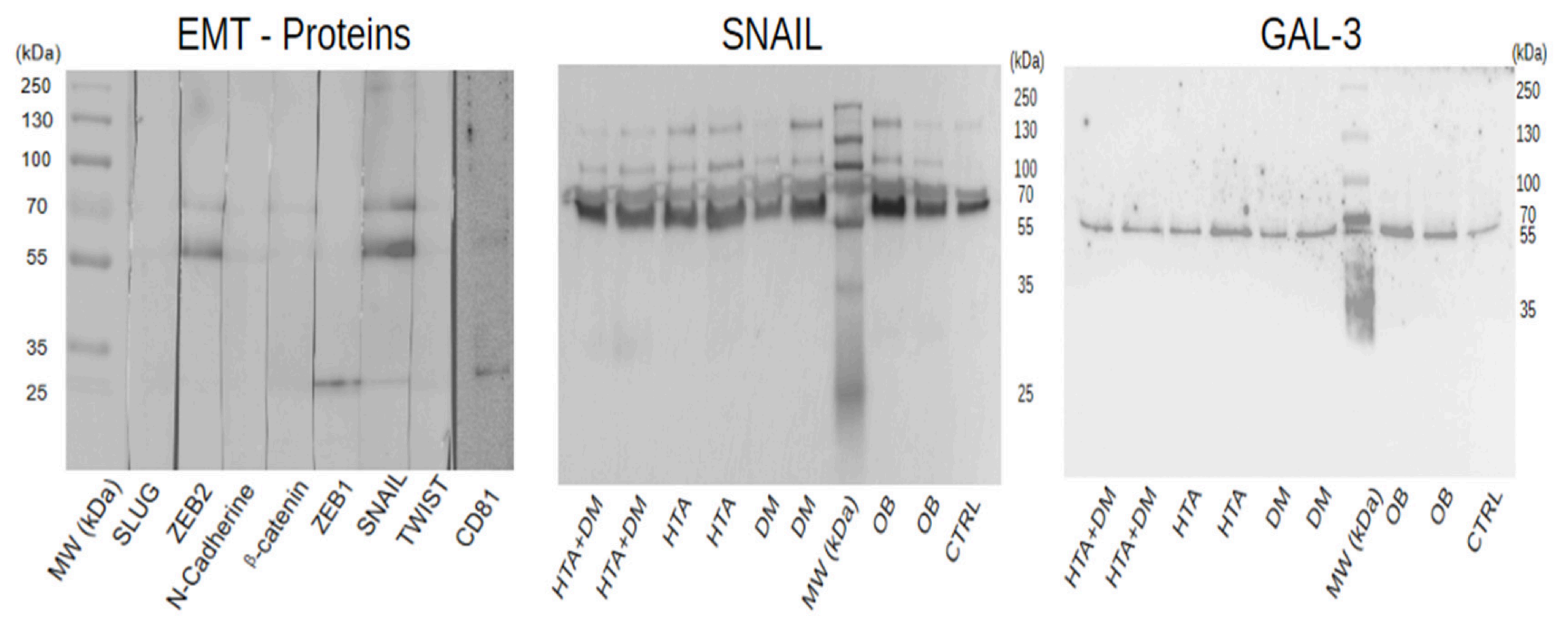
Detection of EMT- and remodeling-associated proteins in plasma-derived extracellular vesicles. Western blot analysis shows EMT-related proteins, SNAIL, and GAL-3 in EVs-enriched preparations from control and cardiometabolic groups. Densitometric analysis was normalized to total protein loading (Amidoblack stain) and expressed relative to the control group. The presented blot represents a single Western blot experiment performed using EVs obtained from two independent isolations per group. Therefore, these findings should be interpreted as semiquantitative, exploratory observations.

As shown in Figure 3, EMT-associated proteins were detected in plasma-derived EVs preparations from both controls and cardiometabolic groups. SNAIL showed the most evident variation across groups, with increased semiquantitative densitometric expression in obesity and hypertension derived EVs compared with controls. which indicated that the primary increase in SNAIL was associated with obesity (1.38-fold) and hypertension (1.16-fold), whereas individuals with diabetes alone or combined hypertension-diabetes showed negligible changes relative to controls (~0.95-fold and ~1.01-fold, respectively).

Galectin-3 (GAL-3) also displayed increased signals across cardiometabolic groups compared with controls, with approximate fold changes of 2.59 in obesity, 1.81 in hypertension, 1.66 in DM, and 1.55 in HTA+DM. Together, these semiquantitative findings suggest that plasma-derived EVs carry proteins linked to inflammation, fibrosis, extracellular matrix remodeling, and endothelial activation.

Following the detection of GAL-3 and SNAIL within the EV preparations, our next objective was to determine whether these vesicles also encapsulate specific miRNA signatures. Given their role in post-transcriptional regulation, evaluating this genetic cargo provides deeper insight into the vesicular mechanisms associated with endothelial dysfunction and cardiometabolic diseases. Because miRNAs constitute a major functional component of extracellular vesicle cargo and can modulate inflammatory, fibrotic, angiogenic, and endothelial signaling pathways, we evaluated whether circulating EVs exhibited disease-associated alterations in their miRNA content [28,29].

### 2.3. Differential miRNA Cargo Profiling in Plasma-Derived Extracellular Vesicles from Individuals with Cardiometabolic Disorders

The detection of EMT-associated proteins within plasma-derived EVs, particularly the increased expression of SNAIL in hypertensive and obesity subjects, prompted the investigation of vesicular microRNAs (miRNAs) as potential regulatory mediators of endothelial dysfunction and cardiometabolic disease.

Preliminary small RNA sequencing analysis identified multiple vesicle-associated miRNAs differentially represented across cardiometabolic conditions (Among the detected candidates, miR-126-3p, miR-21-5p, miR-92a-3p were selected for validation by stem-loop RT-PCR because of their established involvement in endothelial homeostasis, vascular remodeling, inflammation, and atherosclerosis.

**Figure 4:**
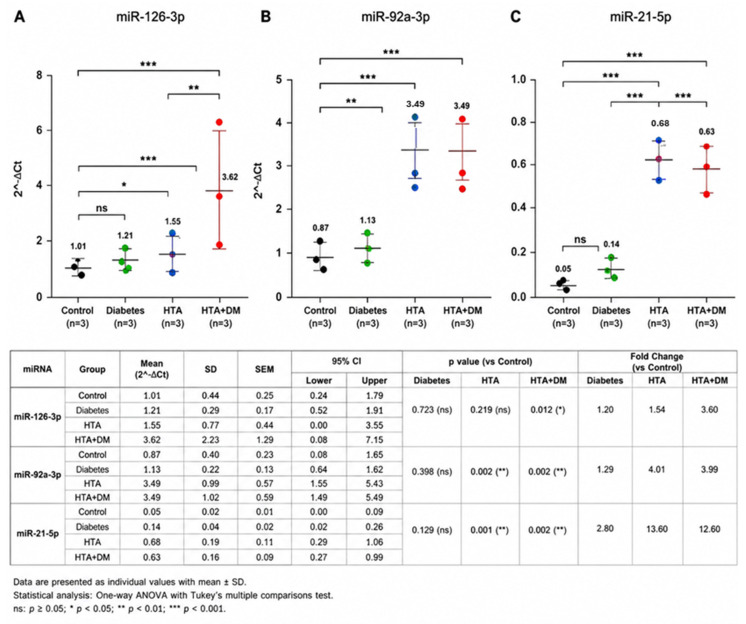
Differential expression of extracellular vesicle-associated miRNAs in plasma-derived small extracellular vesicles from control and cardiometabolic groups. Relative expression levels of selected miRNAs in plasma-derived extracellular vesicles (EVs) isolated from three independent biological samples per clinical group: control subjects (Ctrl) and patients with type 2 diabetes mellitus (DM), hypertension (HTA), and combined hypertension and diabetes mellitus (HTA+DM). Candidate miRNAs were initially identified by small RNA sequencing and subsequently validated by stem-loop RT-PCR. The analyzed targets included (**A**) miR-126-3p, (**B**) miR-92a-3p, (**C**) miR-21-5p. Expression levels were normalized to the endogenous reference control and calculated using the 2^−ΔCt^ method. Individual symbols represent biological samples, and horizontal lines indicate mean ± standard deviation (SD). Statistical comparisons among groups were performed using one-way ANOVA followed by Tukey’s multiple-comparison testing. Significant differences are indicated by asterisks: * *p* < 0.05, ** *p* < 0.01, and *** *p* < 0.001.

As shown in Figure 4, plasma-derived EVs exhibited distinct miRNA expression profiles according to clinical status. Relative expression levels were normalized using endogenous controls and expressed as 2^−ΔCt^ values. The most pronounced differences were observed in hypertensive individuals and in subjects with combined hypertension and diabetes mellitus (HTA+DM).

For miR-126-3p, mean expression increased from 1.01 ± 0.44 in controls to 1.55 ± 0.77 in the HTA group and 3.62 ± 2.23 in the HTA+DM group, corresponding to fold changes of 1.54 and 3.60, respectively. Although no significant differences were observed between controls and diabetic subjects alone (*p* = 0.723) or between controls and HTA (*p* = 0.219), expression was significantly increased in HTA+DM compared with controls (*p* = 0.012).

A more pronounced pattern was observed for miR-92a-3p. Expression levels increased from 0.87 ± 0.40 in controls to 3.49 ± 0.99 in HTA and 3.49 ± 1.02 in HTA+DM, representing approximately four-fold increases relative to controls (fold changes of 4.01 and 3.99, respectively). These differences were statistically significant for both HTA (*p* = 0.002) and HTA+DM (*p* = 0.002), whereas diabetic subjects alone did not differ significantly from controls (*p* = 0.398).

Similarly, miR-21-5p exhibited marked enrichment in vesicles from hypertensive-related groups. Expression increased from 0.05 ± 0.02 in controls to 0.68 ± 0.19 in HTA and 0.63 ± 0.16 in HTA+DM, corresponding to fold changes of 13.60 and 12.60, respectively. Both comparisons reached statistical significance (HTA vs. Control, *p* = 0.001; HTA+DM vs. Control, *p* = 0.002), whereas the increase observed in diabetic subjects alone was not significant (*p* = 0.129).

Collectively, these findings demonstrate that hypertension, either alone or combined with diabetes mellitus, is associated with substantial remodeling of the vesicular miRNA cargo. The concurrent enrichment of miR-126-3p, miR-21-5p, miR-92a-3p suggests the existence of a disease-associated vesicular signature linked to endothelial dysfunction, vascular inflammation, and tissue remodeling. These results further suggest that circulating plasma-derived EVs may serve as biologically active carriers of regulatory miRNAs and represent a potential source of minimally invasive biomarkers for cardiometabolic disease progression.

### 2.4. Evaluation of CD31 (PECAM-1) as EV Marker in an Exploratory Cardiovascular Event Sub-Cohort

**Figure 5:**
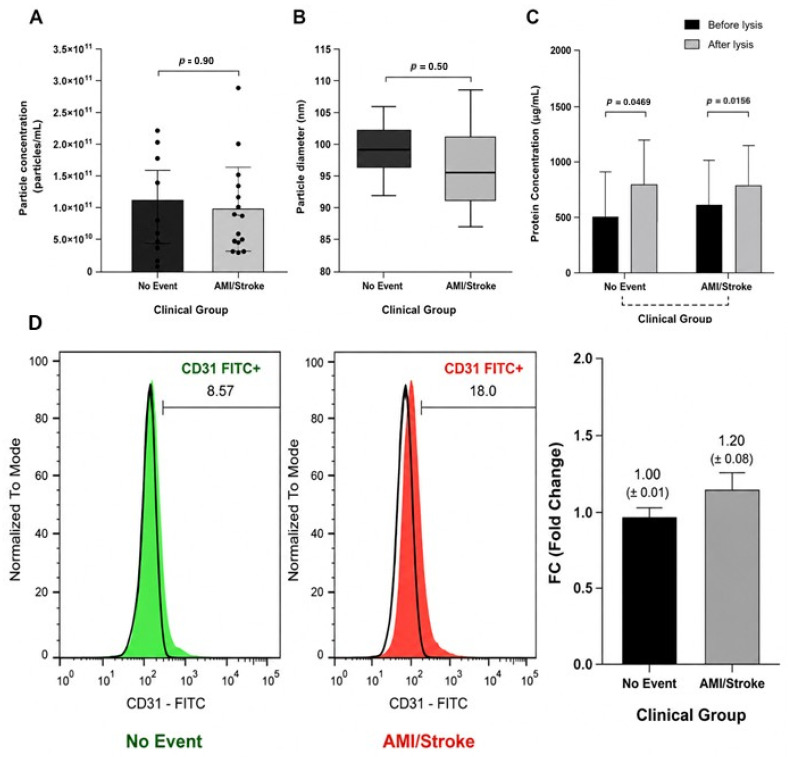
Surface expression of the leukocytes, platelets and endothelial marker CD31 (PECAM-1) in plasma-derived extracellular vesicles from individuals with and without previous cardiovascular events. EVs were captured using anti-CD9 magnetic beads and analyzed by flow cytometry. (**A**) Particle concentration. (**B**) Particle diameter, and (**C**) Protein concentration before and after EVs lysis. (**D**) CD31-FITC histograms and fold-change analysis of CD31 mean fluorescence intensity relative to the no-event group. Data are presented as mean ± SD or median with interquartile range, as appropriate. Statistical comparisons were performed using Mann–Whitney U, Wilcoxon, or Kruskal–Wallis tests with Dunn’s post hoc correction. The standard deviations reported reflect the variation between independent biological pools.

Because SEC-isolated extracellular vesicle (EVs) fractions exhibited increased protein content across cardiometabolic conditions and showed differential miRNA expression profiles, an exploratory characterization of EVs surface markers was performed in a small sub-cohort of selected survivors of major cardiovascular events, including ischemic stroke and acute myocardial infarction (Figure 5). This analysis was undertaken to investigate whether the molecular heterogeneity observed in vesicular cargo was accompanied by differences in markers associated with cellular origin and vascular injury.

Attention was given to CD31 (PECAM-1), a leukocytes, platelets and endothelial-associated marker that has been linked to endothelial activation, vascular damage, and increased cardiovascular risk. Given the central role of endothelial dysfunction in the pathogenesis of obesity, diabetes, hypertension, and atherosclerotic disease, the evaluation of CD31-positive EVs provided additional information regarding the potential endothelial contribution to circulating vesicle populations. The assessment of EVs surface markers, together with vesicular miRNA profiles, help support the concept that distinct EVs subpopulations may be present in different cardiometabolic and cardiovascular conditions. Specifically, in a small cohort of patients (n = 20) from the same population with antecedent of cardiovascular events (AMI or Stroke) vs. patient with cardiometabolic diseases.

Mean Fluorescence intensity (MFI) expressed as fold change relative to the No-event group. Representative histograms demonstrate CD9 positivity and successful EVs capture. (E) Representative histograms of CD81 expression in CD9-captured EVs. CD81 MFI expressed as fold change relative to the No-event group. Data are presented as mean ± SD or median with interquartile range, as appropriate. Statistical comparisons were performed using Mann–Whitney U, Wilcoxon, or Kruskal–Wallis tests with Dunn’s post hoc correction. A *p*-value < 0.05 was considered statistically significant.

Flow cytometric analysis demonstrated successful capture of a CD9-positive EVs subpopulation. Leukocyte-associated markers (CD45, CD80, and CD83) remain below the predefined detection threshold and could not be reliably distinguished from technical background (Supplementary Figure S2). In contrast, CD31 (PECAM-1) was consistently detected in all biological replicates and exhibited a reproducible increase in fluorescence intensity among AMI/stroke survivors compared with subjects without previous cardiovascular events. Although this difference did not reach statistical significance, a relevant trend toward increased CD31 expression was observed (*p* = 0.057).

The efficiency of EV capture was further supported by the high positivity observed for the canonical vesicle markers CD9 and CD81. More than 90% of captured particles were positive for CD81, confirming the enrichment of a CD9^+^/CD81^+^ vesicle population. No significant differences in CD81 fluorescence intensity were observed between clinical groups (*p* = 0.53), although inter-pool variability was evident. Collectively, these findings indicated that the physicochemical properties of circulating EVs were largely comparable between groups, while CD31 emerged as exploratory surface marker showing the most pronounced cardiovascular diseases-associated variation.

### 2.5. Functional Implications of Extracellular Vesicle-Associated miRNAs in Cardiometabolic Disease

**Figure 6:**
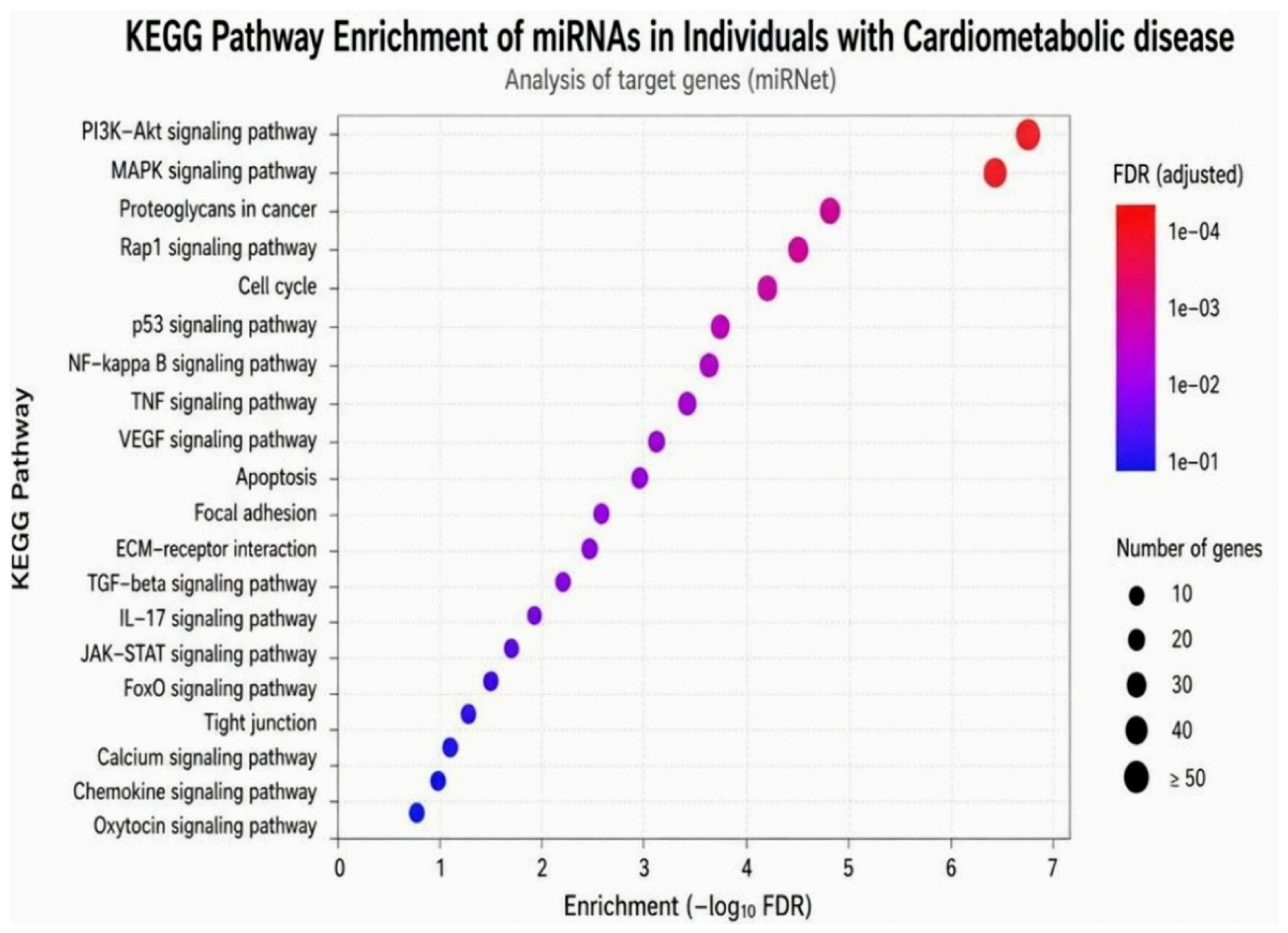
Functional enrichment and miRNA–gene interaction network analysis of extracellular vesicle-associated miRNAs. KEGG enrichment bubble plot generated from predicted and experimentally validated target genes. The *x*-axis represents pathway enrichment as -log10 adjusted false discovery rate (FDR), bubble size corresponds to the number of associated genes, and bubble color indicates adjusted FDR. Enrichment and network analyses were performed using miRNet 2.0 and graphical outputs were refined using Adobe Illustrator 2021 (Version 25.x.). Abbreviations: KEGG, Kyoto Encyclopedia of Genes and Genomes; FDR, false discovery rate; ECM, extracellular matrix; VEGF, vascular endothelial growth factor; TNF, tumor necrosis factor; NF-kB, nuclear factor kappa B.

To investigate the potential biological significance of the altered extracellular vesicle (EV)-associated miRNA signature identified in cardiometabolic conditions, target gene enrichment analysis was performed using experimentally validated and predicted miRNA–mRNA interactions integrated in miRNet 2.0. KEGG and Reactome enrichment analyses revealed significant overrepresentation of signaling pathways involved in vascular homeostasis, inflammation, cell proliferation, apoptosis, and cardiometabolic disease progression (Figure 6).

Among the most significantly enriched pathways were PI3K–Akt, MAPK, Rap1, VEGF, TNF, NF-κB, JAK–STAT, FoxO, p53, TGF-β, and calcium signaling pathways. Additional enrichment was observed for pathways associated with apoptosis, cell cycle regulation, focal adhesion, extracellular matrix (ECM)–receptor interaction, and tight junction signaling. Most pathways remained significant after Benjamini–Hochberg false discovery rate correction, supporting the robustness of the identified miRNA-associated signaling network.

miRNA–gene network analyses further demonstrated extensive interactions between differentially expressed miRNAs and their predicted or validated target genes (Figure 6). Several target genes were connected to more than one miRNA, forming regulatory networks associated with DM, obesity, and hypertension. The integrated network highlighted molecular interactions involved in signal transduction, cellular proliferation, metabolic regulation, and inflammatory responses.

Collectively, these findings indicate that the extracellular vesicle-associated miRNA signature identified in cardiometabolic diseases converges on molecular pathways implicated in endothelial dysfunction, vascular remodeling, inflammation, and metabolic dysregulation.

### 2.6. Clinical and Translational Integration of EVs-Associated Candidates Biomarkers

Integration of epidemiological, anthropometric, metabolic, EV, and bioinformatic findings was performed as a translational synthesis rather than as a single unified predictive model. Within this epidemiological cohort, the TyG index emerged as an independent predictor of elevated WHtR (adjusted OR = 2.76; 95% CI: 1.49–5.12; *p* = 0.001), suggesting a relevant independent association, while hypertension remained independently associated with elevated cardiometabolic risk (adjusted OR = 2.30; 95% CI: 1.05–5.04; *p* = 0.038). Elevated WHtR was also associated with cardiometabolic disease status (adjusted OR = 2.10; 95% CI: 1.50–2.95; *p* < 0.001), supporting the relevance of central adiposity in this population.

At the molecular level, EVs-associated miR-126-3p, miR-21-5p, miR-92a-3p showed the most consistent differences across cardiometabolic groups. Compared with controls, miR-126-3p increased up to 3.60-fold in the HTA+DM group, whereas miR-92a-3p showed approximately four-fold higher expression in both HTA and HTA+DM groups. The largest relative increase was observed for miR-21-5p, which showed 13.6-fold and 12.6-fold elevations in HTA and HTA+DM, respectively.

The CD31-positive EVs marker analysis provided complementary evidence of disease-associated EVs remodeling. Although particle size, particle concentration, and total protein content were not significantly different between cardiovascular event survivors and individuals without previous cardiovascular events, CD31-positive EVs showed increased fluorescence intensity in participants with previous myocardial infarction or stroke, with a trend toward significance (*p* = 0.057). These findings were considered exploratory due to the limited subgroup size.

Bioinformatic analyses showed that the identified EV-associated miRNAs converged on KEGG pathways implicated endothelial dysfunction, inflammation, vascular remodeling, apoptosis, cell-cycle regulation, and extracellular matrix organization, including PI3K-Akt, MAPK, Rap1, VEGF, TNF, NF-kB, JAK-STAT, FoxO, p53, TGF-beta, focal adhesion, and ECM-receptor interaction pathways.

Together, these observations support a conceptual translational framework in which visceral adiposity and insulin resistance could be associated with EVs molecular remodeling, including altered endothelial-associated markers, EMT-related proteins, and disease-associated miRNA cargo. A summary of the integrated clinical and molecular analyses is provided in Supplementary Tables S1–S5.

## 3. Discussion

### 3.1. Cardiometabolic Burden, Endothelial Dysfunction and Extracellular Vesicle Remodeling

The present study provides evidence that plasma-derived extracellular vesicles (EVs) undergo substantial quantitative and qualitative remodeling in individuals with cardiometabolic disorders, particularly hypertension, type 2 diabetes mellitus (DM), and previous cardiovascular events. These findings are particularly relevant because they were obtained in a socially vulnerable population from La Guajira, Colombia, where obesity, insulin resistance, hypertension, dyslipidemia, and limited access to healthcare coexist and contribute to a disproportionately high cardiometabolic burden.

The epidemiological characterization of the cohort demonstrated significant associations between central adiposity, represented by the waist-to-height ratio (WHtR), and metabolic alterations including hypertriglyceridemia, impaired glycemic control, and elevated TyG index. Increasing evidence suggests that visceral adiposity and insulin resistance constitute upstream drivers of endothelial dysfunction and chronic vascular inflammation, two hallmarks of the emerging cardio–hepatic–metabolic syndrome [5,15]. Adipose tissue is now recognized as an active endocrine organ capable of releasing extracellular vesicles enriched in inflammatory mediators, lipids, and regulatory miRNAs that influence distant tissues and contribute to systemic metabolic dysfunction [30,31,32]. Our epidemiological data reinforce the findings of Montoya Castillo et al. (2025) study, which demonstrated that the WHtR (Waist-to-Height Ratio) outperforms TyG in predicting pooled cardiometabolic risks in a large Colombian cohort [33]. This metabolic interaction is important because previous studies have shown that the TyG-WHtR index is independently associated with arterial stiffness. This allows high-risk individuals to be identified in clinical settings [34]. This provides a functional link to the subsequent molecular vascular changes observed in this study.

Within this context, circulating EVs may serve as dynamic indicators of ongoing cellular stress and vascular injury. Because extracellular vesicles are released by virtually all cell types involved in cardiometabolic disease, including endothelial cells, platelets, monocytes, adipocytes, and cardiomyocytes, their molecular cargo can provide an integrated snapshot of the biological processes occurring during disease progression [8,14,16,22,35].

### 3.2. Increased Circulating Vesicle Release in Hypertension Reflects Vascular Stress

There are several challenges when working with EVs from plasma as potential biomarkers, including achieving high-purity isolation. Therefore, we used SEC to isolate EVs, to minimize coprecipitation with lipoproteins, such as LDL and VLDL, which are similar in size and density to small EVs [36,37]. Furthermore, SEC yields plasma isolates that are mostly enriched in EVs while maintaining minimal contamination by plasma proteins [38].

One of the most notable findings of this study was the increase in circulating vesicle abundance observed in hypertensive individuals. Compared with controls, patients with hypertension exhibited significantly higher particle concentrations and fluorescence-positive vesicle populations, with large effect sizes (Figure 1B and Figure 5). This observation is consistent with previous studies demonstrating that chronic hemodynamic stress promotes extracellular vesicle biogenesis through endothelial activation, oxidative stress, inflammatory signaling, and membrane remodeling [15,16,39,40].

Hypertension exposes the vascular wall to persistent mechanical forces that activate endothelial cells and stimulate the release of vesicles enriched in inflammatory and pro-remodeling molecules. Experimental studies have shown that disturbed shear stress, angiotensin II signaling, reactive oxygen species (ROS), and activation of NF-κB pathways all contribute to enhanced EVs production [41,42,43,44]. Accordingly, elevated circulating EVs concentrations have been reported in hypertension, coronary artery disease, metabolic syndrome, and chronic kidney disease, supporting the notion that increased EVs release represents a generalized response to vascular injury [45,46,47,48,49,50].

Interestingly, hypertension showed a major particle concentration compared with the other clinical groups (Figure 1B) that is consistent with increased circulating vesicle release under vascular stress [51]. This study found an increase in EV concentrations in patients with hypertension, consistent with findings from previous studies [52,53]. This may be due to chronically elevated blood pressure subjecting endothelial membranes to non-physiological cyclic stretching. Additionally, in vitro models have demonstrated that changes in blood pressure alter the packing of lipids in cell membranes, accelerating the release of vesicles into circulation [51]. This could explain the results obtained. This finding suggests that endothelial activation induced by hemodynamic stress may represent a potential trigger of EVs release than hyperglycemia itself and reinforces the central role of endothelial dysfunction in the pathogenesis of cardiovascular disease.

### 3.3. Alterations in Vesicular Protein Cargo Could Suggest Disease-Associated Molecular Remodeling

Although total protein concentrations within SEC-derived EVs fractions did not reach statistical significance among groups, a consistent increase was observed in hypertension and hypertension-associated diabetes (Figure 3). This trend could suggest that cardiometabolic disorders affect not only vesicle abundance but also vesicular cargo composition [54,55].

Recent proteomic analyses have demonstrated that extracellular vesicles derived from patients with cardiovascular and metabolic disorders are enriched in proteins involved in coagulation, inflammation, endothelial activation, extracellular matrix remodeling, and fibrosis [56,57]. Consequently, changes in EVs protein content may reflect underlying biological processes occurring within the vascular microenvironment.

The consistent detection of CD81, CD63 and TSG101 confirmed successful enrichment of bona fide extracellular vesicles and validated the isolation strategy according to current MISEV2023 recommendations [58]. Moreover, the presence of higher-molecular-weight bands and multiprotein complexes suggests that plasma-derived EVs may transport structurally organized protein assemblies rather than isolated proteins. Similar observations have been reported in cardiovascular EV proteomics studies and support the concept that EVs function as complex biological signaling platforms capable of coordinating multicellular responses [57]. Overall, the combined NTA, STEM, protein quantification, and immunoblotting results supported successful enrichment of plasma-derived EVs. However, as expected for plasma EVs preparations, the possible contribution of co-isolated soluble proteins, lipoproteins, and protein complexes cannot be completely excluded. Finally, these results indicated that cardiometabolic conditions may be associated with changes in EVs-associated protein cargo rather than uniform differences in total vesicle yield. Accordingly, subsequent analyses focused on whether these EVs-enriched preparations carried proteins related to tissue remodeling and endothelial dysfunction.

### 3.4. SNAIL-Positive Extracellular Vesicles Suggest Activation of EMT/EndMT-Associated Pathways

A particularly novel observation was the detection and enrichment of SNAIL within circulating EVs derived from hypertensive and a moderate increase in obesity individual. To our knowledge, relatively few studies have investigated the presence of EMT/EndMT-associated transcription factors within plasma-derived extracellular vesicles in cardiometabolic disease.

SNAIL is a master regulator of epithelial-to-mesenchymal transition (EMT) and endothelial-to-mesenchymal transition (EndMT), processes that have emerged as critical contributors to fibrosis, vascular stiffening, atherosclerosis, diabetic vasculopathy, and chronic kidney disease [26,27,59]. Under conditions of chronic metabolic stress, activation of TGF-β, NF-κB, and oxidative stress pathways promotes SNAIL expression and initiates transcriptional programs associated with cellular plasticity, extracellular matrix deposition, and inflammatory remodeling [60,61,62,63]. The presence of SNAIL in circulating vesicles suggests a much broader paradigm regarding cell communication mediated by EVs. To our knowledge, SNAIL has only previously been reported in the work of You et al. (2019) [26]. Regarding GAL-3, it has previously been detected in patients with decreased ventricular function, which could explain its presence in the evaluated groups Clementy et al., 2018 [64].

The detection of the EndMT master regulator SNAIL within circulating EVs is a novel observation in the context of cardiometabolic stress, suggesting that EVs may reflect cellular plasticity events occurring in the vascular wall. Importantly, our findings do not demonstrate that SNAIL-containing EVs directly induce EndMT in recipient cells [65,66]. Rather, they could suggest that EV cargo mirrors molecular events associated with endothelial dysfunction and tissue remodeling.

Given the recognized role of EndMT in cardiovascular disease progression, the identification of SNAIL within plasma-derived EVs represents a potentially important finding that warrants validation in larger cohorts and mechanistic studies. Furthermore, given the documented role of GAL-3 in chronic inflammation and tissue fibrosis, its detection within the evaluated clinical samples (Figure 3) highlights the potential physiological relevance of these vesicle profiles. Collectively, the exploratory detection of SNAIL, GAL-3, and canonical vesicle markers supports the presence of biologically active protein cargo within plasma-derived EV preparations.

### 3.5. Remodeling of Vesicular miRNA Cargo Reflects Endothelial Dysfunction and Vascular Injury

The miRNA analyses further support the concept that circulating extracellular vesicles function as biologically active carriers of regulatory information. Among all identified candidates, miR-126-3p emerged as the most consistently enriched miRNA within vesicles derived from cardiometabolic patients [67,68,69].

miR-126 is widely considered an endothelial-enriched miRNA that regulates angiogenesis, endothelial repair, vascular integrity, VEGF signaling, and PI3K-Akt pathway activation [70,71,72]. Alterations in circulating miR-126 levels have been associated with hypertension, diabetes, coronary artery disease, and endothelial dysfunction. Several studies have suggested that EV-associated miR-126 may serve as an adaptive response aimed at preserving vascular homeostasis following endothelial injury [69,73].

In contrast, the increased abundance of miR-21-5p and miR-92a-3p supports activation of inflammatory and remodeling pathways. miR-21 is strongly linked to TGF-β signaling, fibrosis, and extracellular matrix accumulation, whereas miR-92a suppresses endothelial-protective genes such as KLF2 and KLF4 and contributes to endothelial dysfunction and atherogenesis [74,75,76].

The simultaneous enrichment of miR-126-3p, miR-21-5p, miR-92a-3p, and SNAIL transcripts suggest the existence of a coordinated vesicular response balancing endothelial repair, inflammation, vascular remodeling, and fibrosis [77,78,79,80]. This integrated molecular signature may therefore provide more informative insights than individual biomarkers considered in isolation.

### 3.6. CD31-Positive Extracellular Vesicles as Exploratory Indicator of Endothelial Injury and Residual Cardiovascular Risk

The evaluation of EVs surface markers identified CD31 (PECAM-1) as the marker showing relevant disease-associated variation (Table 2). Although the difference did not reach statistical significance, likely due to the limited sample size of the cardiovascular event sub cohort, AMI and stroke survivors consistently exhibited a moderate increased CD31 expression than subjects without previous cardiovascular events.

CD31 is a transmembrane glycoprotein highly expressed by leukocytes, platelets and endothelial cells, where it regulates vascular integrity, Mechan sensing, leukocyte transmigration, angiogenesis, and inflammatory responses [81,82,83,84,85]. Elevated levels of circulating CD31-positive vesicles have been reported in hypertension, diabetes, obesity, dyslipidemia, subclinical atherosclerosis, acute coronary syndromes, and vascular aging [81,82,86]. Finally, the trend towards an increase in CD31-positive EVs (PECAM-1) in patients with a history of acute ischemic events, such as myocardial infarction or stroke (*p* = 0.057), is consistent with previous evidence indicating that this marker can be useful for predicting the risk of cardiovascular events [23] [Ref].

Importantly, no significant differences were observed in vesicle size, concentration, total protein content, or CD81 expression between cardiovascular-event survivors and non-event subjects. This observation could suggest that disease-associated information is primarily reflected by vesicle composition rather than by absolute vesicle abundance. Consequently, endothelial-derived EVs may represent more sensitive indicators of residual vascular injury than traditional particles count alone.

**Table 2:** Vesicular Markers According to Cellular Origin, Cardiovascular Risk Factors, and Other Clinical Associations.

Condition	Vesicular Marker	Reference
Cell of Origin		
Lymphocytes	CD3, CD45, CD28	[87,88]
Monocytes	CD11b, CD14, CD31, CD64, CD142	[89,90,91]
Polymorphonuclear cells	CD35, CD66b, CD11	[92,93]
Platelets	CD31, CD41, CD42b, CD61	[94,95]
Endothelium	CD144, CD31, CD106, CD62E, CD54	[96,97,98]
Cardiomyocytes	HSP20, HSP70, HSP60	[99,100]
Cardiovascular Risk Factor		
Hypertension	CD31, CD41, CD42	[101,102,103,104]
Diabetes	CD66, CD51, CD31, CD41	[105,106,107,108]
Obesity/Overweight	CD31, CD62E	[109,110,111]
Dyslipidemia	CD31, CD144, CD62E	[110,111,112,113]
Smoking	CD41, CD45	[114]
Stages of the Atherosclerotic Plaque		
Subclinical plaque	CD105, CD11a	[115]
Stable plaque	CD31, CD144, CD42	[116,117]
Unstable plaque	CD31, CD42, CD11b, CD66, CD15	[118]
Acute coronary syndrome plaque	CD31, CD146, CD42, CD11a, CD3, CD235a	[119,120]

Abbreviations: CD, cluster of differentiation; HSP, heat shock protein. Note: Surface markers identified in circulating extracellular vesicles have been associated with specific cellular origins, cardiovascular risk factors, and stages of atherosclerotic disease progression.

### 3.7. Integrated Molecular Signature Supports an Endothelium–EVs–miRNA–Remodeling Axis

A major contribution of this study is the convergence of epidemiological and molecular observations toward a common framework centered on endothelial dysfunction. Increased vesicle release, enrichment of miR-126-3p, miR-21-5p and miR-92a-3p, detection of SNAIL, and increased CD31-positive EVs collectively support the presence of an endothelial stress-related EV signature in cardiometabolic disease.

**Figure 7:**
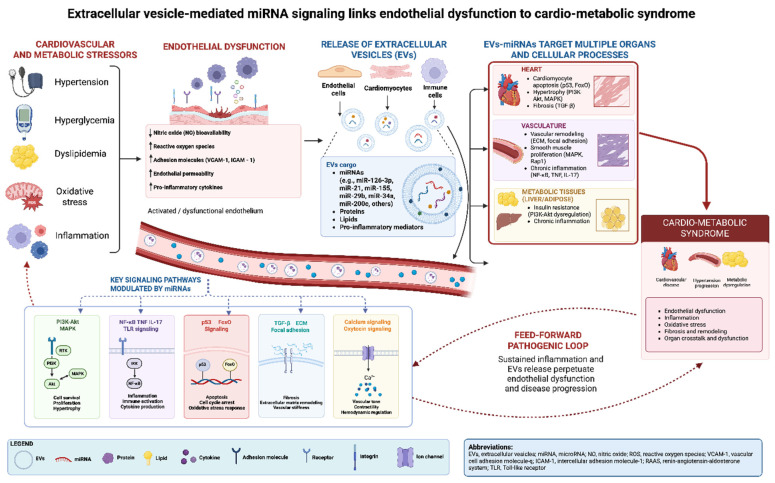
Proposed mechanism linking EVs to cardiometabolic syndrome. Hypothetical and literature-derived models integrate cardiometabolic stress, EV miRNA cargo, and endothelial dysfunction into a coordinated network governing vascular injury. Functional enrichment analysis revealed significant associations with key pathways, including PI3K–Akt, MAPK, VEGF, TNF, NF-κB, TGF-β, JAK–STAT, FoxO, p53, Rap1, calcium signaling, apoptosis, focal adhesion, and ECM–receptor interactions. Together, variations in vesicle abundance, CD31-positive EVs, SNAIL expression, and specific miRNA signatures indicate that extracellular vesicles serve as both biomarkers and active drivers of endothelial dysfunction and cardiometabolic progression. Abbreviations: EVs, small extracellular vesicles; ROS, reactive oxygen species; NO, nitric oxide; EMT, epithelial-to-mesenchymal transition; EndMT, endothelial-to-mesenchymal transition; ECM, extracellular matrix; PI3K, phosphoinositide 3-kinase; MAPK, mitogen-activated protein kinase; VEGF, vascular endothelial growth factor; TNF, tumor necrosis factor; NF-κB, nuclear factor kappa B; JAK–STAT, Janus kinase/signal transducer and activator of transcription. Created with biorender.com (Aulestia, M., 2026) and adapted using experimental findings from the present study and relevant literature.

Accordingly, we propose an exploratory endothelium-EV-miRNA-remodeling axis in which cardiometabolic stress is associated with endothelial activation and selective EVs cargo remodeling (Figure 7). This model should be considered hypothesis-generating and requires validation in longitudinal and mechanistic studies.

### 3.8. Clinical and Translational Implications of EVs-Associated Potential Biomarkers in Cardio-Metabolic Diseases

The translational framework represents a hypothesis-generating integrative interpretation and should not be considered a predictive model validated at the individual level. The integration of epidemiological, anthropometric, metabolic, extracellular vesicle, and bioinformatic findings provides a biologically plausible framework linking cardiometabolic risk factors with molecular processes involved in endothelial dysfunction and cardiovascular disease progression. Although molecular analyses were performed in selected samples and several miRNA measurements were derived from pooled specimens, the findings showed consistency across independent analytical levels. Enrichment of EVs-associated miR-126-3p, miR-92a-3p, and miR-21-5p paralleled the epidemiological evidence that central adiposity and insulin resistance, represented by WHtR and TyG, could suggest a possible association with cardiometabolic risk.

Among the identified candidates, miR-126-3p deserves particular attention because of its established role in endothelial homeostasis, angiogenesis, vascular repair, and maintenance of endothelial integrity. Increased levels of vesicular miR-126-3p have been reported in conditions characterized by endothelial activation and vascular stress, suggesting that its elevation may reflect a compensatory response to ongoing endothelial injury [67,71]. Likewise, miR-92a-3p has been consistently associated with endothelial dysfunction, impaired angiogenic responses, and atherosclerotic progression, whereas miR-21-5p is recognized as a major regulator of inflammation, fibrosis, vascular remodeling, and tissue repair [80,121,122]. The simultaneous enrichment of these miRNAs in hypertensive and hypertensive-diabetic individuals supports the existence of coordinated regulatory networks involved in vascular adaptation and chronic cardiometabolic stress.

The biological relevance of this vesicular miRNA signature is reinforced by the pathway enrichment analyses. Differentially expressed EV-associated miRNAs converged on signaling pathways critically implicated in cardiometabolic disease pathogenesis, including PI3K–Akt, MAPK, Rap1, VEGF, NF-κB, TNF, JAK–STAT, FoxO, p53, and TGF-β signaling. These pathways collectively regulate endothelial function, inflammatory responses, extracellular matrix remodeling, apoptosis, cellular senescence, and vascular integrity. Importantly, dysregulation of these signaling networks has been extensively linked to hypertension, diabetes mellitus, atherosclerosis, chronic kidney disease, and heart failure, supporting the biological plausibility of the identified vesicular biomarkers as indicators of ongoing vascular injury and remodeling.

An additional finding that strengthens the endothelial component of the proposed model is the increased expression of CD31-positive EVs observed among individuals with a history of myocardial infarction or stroke. Although this difference did not reach conventional statistical significance, likely due to the limited size of the exploration cardiovascular cohort, the consistency of the observed trend suggests enhanced release of endothelial-derived vesicles in subjects with established vascular disease. This interpretation is supported by previous studies reporting elevated circulating endothelial EVs in atherosclerosis, coronary artery disease, cerebrovascular disease, and other conditions characterized by endothelial injury and vascular dysfunction. Notably, the absence of significant differences in particle size, particle concentration, or total vesicular protein content between groups suggests that disease-associated alterations may be driven primarily by changes in EV cellular origin and molecular cargo rather than by overall vesicle abundance.

The concurrent detection of SNAIL within circulating EVs provides an additional mechanistic link between endothelial dysfunction and tissue remodeling. SNAIL is a master regulator of epithelial-to-mesenchymal transition (EMT) and endothelial-to-mesenchymal transition (EndMT), processes increasingly recognized as contributors to fibrosis, vascular remodeling, and chronic organ dysfunction. The enrichment of SNAIL together with endothelial-associated miRNAs supports the hypothesis that circulating EVs may participate in systemic signaling networks connecting metabolic stress, inflammation, endothelial injury, and structural remodeling of cardiovascular tissues.

From a translational perspective, the present findings suggest that EV-associated biomarkers may complement conventional clinical risk assessment tools. Current clinical practice relies primarily on conventional risk factors, biochemical markers, and imaging approaches to identify cardiovascular risk. However, these tools frequently detect disease only after substantial vascular damage has already occurred. In contrast, extracellular vesicles provide direct access to ongoing molecular processes occurring at the cellular level and may therefore enable earlier detection of endothelial dysfunction and cardiometabolic deterioration [14,54,120,123]. The integration of anthropometric indicators such as WHtR, metabolic indices such as TyG, EVs carrying associated marker CD31, EMT-associated proteins such as SNAIL, and EV-associated miRNAs may therefore offer a multidimensional and biologically informed approach for identifying individuals at increased cardio–metabolic risk.

Importantly, the combined assessment of CD31-positive EVs, miR-126-3p, miR-21-5p, miR-92a-3p, and SNAIL may represent a promising exploratory liquid-biopsy signature for future validation. Unlike single biomarkers, this multiparametric panel captures endothelial injury, inflammation, vascular remodeling, and EMT/EndMT-associated processes within a single biological framework. Its diagnostic, prognostic, and therapeutic-response value should be tested in independent prospective cohorts.

Taking together, these findings support the concept that extracellular vesicles may reflect intercellular communication processes linking visceral adiposity, insulin resistance, endothelial dysfunction, inflammation, and tissue remodeling. The integrated signature composed of CD31-positive EVs, miR-126-3p, miR-21-5p, miR-92a-3p, and SNAIL could be considered a possible candidate biomarker panel that requires additional validation before clinical application.

### 3.9. Limitations and Future Perspectives

Several limitations should be considered when interpreting these findings:

Detailed information regarding pharmacological treatments was not consistently available for all participants, preventing its systematic inclusion in the multivariable analysis. This represents a limitation, as certain medications are known to modulate EVs secretion and cargo, potentially introducing residual confounding. However, to mitigate this limitation and reduce unmeasured biological variability, strict inclusion criteria were applied during the study design. The cohort was restricted to a narrow age range (51–64 years) and a specific ethnic background (non-Indigenous and non-Afro-Colombian). More importantly, we selected only individuals reporting a single comorbidity, which inherently limited the diversity and burden of concurrent drug regimens. This phenotypic homogeneity partially offsets the lack of pharmacological data and minimizes the confounding influence of complex therapies on EV production, thereby strengthening the internal validity of our findings.

The cross-sectional design precludes causal inference; consequently, the observed associations between EV cargo and clinical status are correlational and do not imply disease progression or causality. Furthermore, in the absence of functional experiments, the biological pathways and regulatory roles inferred from bioinformatic enrichment analyses remain putative and should be regarded as exploratory and hypothesis-generating frameworks.

Despite the use of SEC effectively reducing plasma protein contamination, the co-isolation of high-density lipoproteins (HDL, LDL) and other soluble protein complexes cannot be entirely excluded and may contribute to the observed protein signals in Western blots.

Several molecular analyses were performed using pooled plasma samples, a strategy adopted to maximize the use of limited biological material and facilitate exploratory screening of extracellular vesicle cargo. Although this approach allowed the identification of consistent molecular patterns across clinical groups, it limited the assessment of interindividual variability and precluded individual-level inference. Similarly, validation of selected vesicular miRNAs was performed in a relatively small number of biological replicates, and therefore the results should be considered hypothesis-generating. Finally, pooled samples do not allow assessment of interindividual variability; technical replicates were never treated as biological replicates; statistical inference should therefore be interpreted cautiously.

An additional limitation relates to the small RNA sequencing analysis. The sequencing experiment should be considered a discovery-oriented screening approach rather than a comprehensive characterization of the vesicular miRNome. Although approximately 10 million sequencing reads were generated and passed standard quality-control criteria, the number of annotated mature miRNAs identified within the EV preparations was relatively limited. This likely reflects the intrinsically low RNA content of plasma-derived EVs, the low abundance of mature miRNAs within circulating vesicles, and the presence of other small RNA species competing during library preparation and sequencing. Furthermore, the combination of pooled samples and moderate sequencing depth may have reduced the sensitivity to detect low-abundance miRNAs and subtle differences between clinical groups. Consequently, the sequencing results should be regarded primarily as an exploratory discovery dataset intended to identify candidate miRNAs for subsequent validation rather than as a comprehensive characterization of the entire vesicular miRNA repertoire. Importantly, the most biologically relevant candidates identified by sequencing were independently validated by stem-loop RT-qPCR, strengthening the robustness of the principal molecular findings.

The precise cellular origin of CD31-positive EVs was not established, and the exploratory analysis of CD31-positive EVs was conducted in a relatively small subgroup of cardiovascular event survivors and therefore lacked sufficient statistical power to draw definitive conclusions regarding endothelial-derived vesicle populations. The observed trend toward increased CD31 expression in subjects with previous myocardial infarction or stroke should therefore be interpreted cautiously and require confirmation in larger independent cohorts.

Finally, the present study did not include mechanistic or functional experiments to determine whether the identified EV-associated miRNAs, proteins, or endothelial markers directly influence endothelial activation, oxidative stress, inflammation, vascular permeability, fibrosis, or endothelial-to-mesenchymal transition (EndMT). Therefore, the biological functions inferred from pathway enrichment and network analyses remain putative and require experimental validation.

Despite these limitations, the convergence of epidemiological, anthropometric, metabolic, extracellular vesicle, molecular, and bioinformatic findings across multiple analytical platforms provides a coherent and biologically plausible framework linking visceral adiposity, insulin resistance, endothelial dysfunction, extracellular vesicle remodeling, and cardiovascular disease. Future studies should incorporate larger prospective cohorts, individual-level multi-omics analyses, deeper small RNA sequencing, high-resolution single-vesicle phenotyping, proteomic characterization, and mechanistic experiments to validate the causal role of EV-associated cargo in disease progression and to assess its clinical utility as a biomarker.

Collectively, the present findings support the concept that plasma-derived extracellular vesicles constitute a rich source of information regarding endothelial injury and cardiometabolic dysfunction and identify miR-126-3p, miR-21-5p, miR-92a-3p, SNAIL, and CD31-positive EVs could be promising candidate biomarkers for future translational and precision-medicine applications in cardio–metabolic disease.

## 4. Materials and Methods

### 4.1. Study Population and Clinical Stratification

This study was conducted within the framework of a larger cross-sectional analytical investigation designed to characterize cardiometabolic risk factors in adults residing in the municipalities of Riohacha, Maicao, and Manaure, in the department of La Guajira, Colombia. The parent cohort comprised 602 participants recruited through community-based outreach programs coordinated by healthcare institutions, non-communicable disease (NCD) prevention programs, and local community leaders. The epidemiological characterization of this population, including the associations between central adiposity, metabolic biomarkers, and cardiometabolic risk factors, has been reported separately and served as the basis for the selection of participants included in the extracellular vesicle (EV) analyses.

Sociodemographic, medical, anthropometric, and biochemical examinations were performed on consenting adults (≥18 years). The anthropometric analysis comprised of the measurement of WHtR (waist-to-height ratio) as an index of central obesity. Blood samples collected from fasting subjects were assessed for biochemical parameters such as glucose, lipid profile, homocysteine, and vitamin B12 levels while the TyG index was calculated as a surrogate measure of insulin resistance. Multivariate logistic regression analyses were conducted where elevated WHtR was used as the dependent variable, with the TyG index being the independent variable.

For the present study, participants were clinically classified according to the presence or absence of cardiometabolic and cardiovascular conditions into the following groups: healthy controls without a history of non-communicable chronic diseases (Ctrl), obesity (OB), hypertension (HTA), type 2 diabetes mellitus (DM), combined hypertension and type 2 diabetes mellitus (HTA+DM), previous acute myocardial infarction (AMI), and ischemic stroke. This stratification was subsequently used for plasma-derived small extracellular vesicle isolation and molecular analyses aimed at identifying EV-associated biomarkers related to endothelial dysfunction, cardiovascular disease, and cardio–metabolic risk.

For the molecular analyses, a matched sub-cohort was selected to reduce biological heterogeneity, the analytical cohort was restricted to women aged 51–64 years, with either healthy status or a single cardiometabolic morbidity, excluding men, participants with multimorbidity, individuals outside the target age range, and those self-reporting Indigenous or Afro-Colombian ethnicity. This strategy generated a clinically comparable sub-cohort suitable for exploratory extracellular vesicle and molecular profiling. Participants were stratified into five groups: healthy controls, hypertension, type 2 diabetes mellitus, obesity, and combined hypertension plus type 2 diabetes mellitus. Representative samples from each group were organized into pools to obtain sufficient biological material, reduce inter-individual variability, and enable cost-effective exploratory screening of extracellular vesicle-associated molecular markers. The pooling approach was intended to identify candidate disease-associated molecular patterns, which should subsequently be confirmed through individual-level validation in samples from each clinical group. This two-step strategy allows initial discovery under controlled clinical conditions, followed by assessment of reproducibility, biological variability, and potential biomarker relevance at the individual participant level.

The study was conducted in accordance with the Declaration of Helsinki and was approved by the Ethics Committee of the Colombian National Institute of Health (CEMIN-16-2023, Act No. 21, 14 July 2023). Written informed consent was obtained from all participants prior to enrollment.

### 4.2. Isolation and Characterization of Plasma-Derived Small Extracellular Vesicles

Peripheral blood samples were collected in EDTA tubes and processed within 2 h of collection. Plasma was obtained by sequential centrifugation at 2500× *g*. Prior to SEC separation, the plasma sample was centrifuged for 15 min, followed by 10,000× *g* for 30 min and 19,000× *g* for 30 min at 4 °C to remove residual cellular debris, apoptotic bodies, platelets, and larger extracellular vesicles.

Extracellular vesicles were subsequently isolated using qEVoriginal Gen 2 SEC columns with a nominal 35 nm pore size (qEV1 Gen2/35 nm; IZON Science Ltd., Christchurch, New Zealand) according to the manufacturer’s recommendations. Prior to sample loading, columns were equilibrated with sterile phosphate-buffered saline (PBS; Gibco, Thermo Fisher Scientific, Waltham, MA, USA). A volume of 500 μL of plasma was loaded onto each column, and vesicle-enriched fractions were collected sequentially. Based on the elution profile established during method standardization, the four fractions corresponding to the extracellular vesicle-rich zone were collected (400 μL each). For downstream analyses, selected fractions were pooled and stored at −80 °C until characterization.

For protein and molecular analyses requiring sample concentration, pooled SEC fractions were concentrated using Amicon Ultra-4 centrifugal filter units with a 10 kDa molecular weight cut-off (MilliporeSigma, Burlington, MA, USA). Vesicle preparations were processed according to the Minimal Information for Studies of Extracellular Vesicles (MISEV) recommendations [58].

Isolated vesicles were characterized using complementary approaches, including nanoparticle tracking analysis (NTA), protein quantification, immunoblotting, electron microscopy, and surface marker profiling, to confirm their size distribution, morphology, and molecular composition. We have submitted all relevant data of our experiments to the EV-TRACK knowledgebase (EV-TRACK ID: EV260064, Gómez-Grosso (Author last name)) [124].

#### 4.2.1. Isolation of EVs for Methodological Validation (Ultracentrifugation Control)

To serve as a methodological reference control and validate the size distribution profile of the isolated vesicles, a subset of plasma samples was processed using a standard differential ultracentrifugation protocol. Briefly, plasma was centrifuged sequentially at 10,000× *g* and 19,000× *g* to remove larger vesicles and cellular debris. The supernatant was then subjected to ultracentrifugation at 100,000× *g* for 2 h at 4 °C. The resulting EV pellet was resuspended in PBS, stored at –80 °C, and subsequently washed with 1X PBS prior to Nanoparticle Tracking Analysis (NTA) comparison with the size-exclusion chromatography (SEC) fractions.

#### 4.2.2. Nanoparticle Tracking Analysis

Particle size distribution and concentration were determined by nanoparticle tracking analysis (NTA) using a NanoSight NS300 instrument (Malvern Panalytical, Malvern, UK). Vesicle suspensions were diluted in sterile filtered PBS to achieve the optimal particle concentration recommended by the manufacturer. Five independent videos of 60 s each were recorded under identical acquisition settings for every sample. Videos were analyzed using NanoSight NTA software (version 3.4), and mean particle diameter, modal size, and particle concentration were calculated. Results were expressed as particle diameter (nm) and particles/mL [11,125,126].

#### 4.2.3. Protein Quantification and Western Blot Analysis

Total protein concentration in SEC-derived vesicle preparations was determined using the Bradford colorimetric assay (Quick Start™ Bradford 1× Dye Reagent; Bio-Rad Laboratories, Hercules, CA, USA) according to the manufacturer’s instructions.

For Western blot analysis, equal amounts of vesicular protein (approximately 15 µg) were separated by 10% SDS–polyacrylamide gel electrophoresis and transferred onto PVDF membranes. Membranes were blocked using polyvinylpyrrolidone (PVP40) for 2 h at room temperature and membranes were incubated with primary antibodies against the canonical extracellular vesicle markers CD63 (ab59479, Abcam, Cambridge, UK), CD81 (ab79559, Abcam, Cambridge, UK), and TSG101 (ab83, Abcam, Cambridge, UK). Additional immunoblot analyses included epithelial–mesenchymal transition (EMT)-related proteins, including β-catenin (sc-7199), N-cadherin (sc-7939), SNAIL (sc-28199), SLUG (sc-15391), TWIST (sc-15393), ZEB1 (sc-25388), and ZEB2 (sc-48789) (Santa Cruz Biotechnology, Dallas, TX, USA). Finally, membranes were incubated with horseradish peroxidase-conjugated secondary antibodies, and immunoreactive bands were visualized using the ECL Prime Western Blotting Detection Kit (Amersham Biosciences, Buckinghamshire, UK) and captured using a ChemiDoc™ Imaging System (Bio-Rad Laboratories, Hercules, CA, USA). Densitometric analyses were performed using membrane-stained amidoblack with 0.1% (*w*/*v*) Amido black in 45% methanol and 10% acetic acid [127] and estimation of total protein concentration was determined with Image Lab software version 6.0 (Bio-Rad Laboratories).

#### 4.2.4. Scanning Transmission Electron Microscopy

The morphology and ultrastructural characteristics of isolated vesicles were evaluated by scanning transmission electron microscopy (STEM). Vesicle suspensions were deposited onto carbon-coated copper grids and incubated for 15 min to allow particle adsorption. Excess liquid was removed using filter paper, and samples were negatively stained with 3% uranyl acetate. Grids were air-dried and examined using a Lyra 3 scanning electron microscope equipped with a transmitted electron detector (R-STEM; Tescan Orsay Holding, Brno, Czech Republic) operating at an accelerating voltage of 30 kV [128,129].

Representative micrographs were acquired at multiple magnifications to evaluate vesicle morphology, integrity, and size distribution. Vesicles displaying the characteristic spherical or cup-shaped morphology and nanometric dimensions expected for extracellular vesicles were considered consistent with EVs preparations.

#### 4.2.5. Flow Cytometric Characterization of Small Extracellular Vesicle Surface Markers

To further investigate the cellular origin and biological heterogeneity of circulating small extracellular vesicles (EVs), an exploratory surface marker characterization was performed using bead-assisted flow cytometry. This analysis was motivated by the increased protein content observed in size-exclusion chromatography (SEC)-isolated vesicle fractions and the differential expression of vesicle-associated microRNAs identified across cardiometabolic conditions. Emphasis was placed on CD31 (PECAM-1), a leukocytes, platelets and endothelial-associated marker previously linked to endothelial activation, vascular injury, atherosclerosis, and increased cardiovascular risk.

Due to limited sample availability and the exploratory nature of the study, a sub-cohort of individuals was used. These individuals were distributed into 3 independent pools for the non-event group (without AMI or ischemic stroke) and 4 independent pools for the AMI/Stroke group. Marker analyses were performed according to sample availability; therefore, some markers were evaluated in all pools, whereas others were assessed only in a subset of samples.

Plasma-derived extracellular vesicles were initially enriched by differential centrifugation. Briefly, plasma samples were centrifuged at 10,000× *g* for 30 min, and the resulting supernatant was subsequently centrifuged at 17,000× *g* for 70 min. The final supernatant was collected and stored at −80 °C until analysis. For flow cytometric characterization, extracellular vesicles were captured using the ExoStep™ Plasma + Lyophilized Exosome Kit (Cat. No. ExoS-50-PST81, ImmunoStep, Salamanca, Spain) according to the manufacturer’s instructions with minor modifications. This assay employs anti-CD9-coated magnetic capture beads and therefore selectively enriches the CD9-positive sEV subpopulation. Additionally, a single exploratory experiment was performed using the ExoStep™ Cell Culture + Lyophilized Exosome Kit (Cat. No. ExoS-50-CST9, ImmunoStep, Salamanca, Spain), which utilizes anti-CD63-coated beads.

For vesicle capture, 45 μL of incubation buffer and 5 μL of anti-CD9 capture beads were mixed with 50 μL of sample in low-binding microcentrifuge tubes to minimize vesicle loss by adsorption to tube surfaces. Samples were incubated overnight at 4 °C in the dark without agitation. Following incubation, beads were washed with an assay buffer and recovered by centrifugation.

Captured vesicles were subsequently immunoassayed with fluorochrome-conjugated antibodies directed against vesicle-associated and cell lineage-associated markers, including CD81, CD31 (Clone WM59, BD Biosciences), CD45 (Clone J33, Beckman Coulter), CD80 (BioLegend), and CD83. CD31 was selected as the primary marker of interest because of its recognized association with endothelial dysfunction and cardiometabolic disease. Additional exploratory analyses included HLA-DR and apolipoprotein E (APOE). Antibody incubations were performed for 60 min at 4 °C in the dark without agitation. Following staining, samples were washed with an assay buffer, collected by centrifugation, and resuspended in an assay buffer until acquisition.

Several controls were included to establish assay specificity and fluorescence thresholds, including capturing beads alone, beads incubated with plasma in the absence of antibodies, and beads incubated with antibodies only. During assay standardization, a lyophilized exosome standard provided by the manufacturer (human plasma-derived and PC3 cell-derived exosomes) was used in combination with anti-CD81 antibody as a positive control to validate assay performance. All controls were processed under identical experimental conditions.

Data acquisition was performed on a BD FACSMelody™ flow cytometer (BD Biosciences, San Jose, CA, USA) using at least technical duplicates for each sample. Data were analyzed using FlowJo™ v10 software (BD Biosciences, Ashland, OR USA). The gating strategy consisted of initial identification of the bead population based on forward scatter (FSC) and side scatter (SSC) characteristics, followed by doublet exclusion using FSC-A versus FSC-H plots. Subsequently, events positive for both APC and PerCP-Cy5.5 fluorescence, corresponding to the capture beads according to the manufacturer’s specifications, were selected for downstream analysis.

Marker positivity was quantified as the proportion of fluorescent events above a threshold established from the corresponding negative controls. To distinguish true biological signal from background fluorescence, a conservative positivity threshold was defined as the mean fluorescence intensity (MFI) of the negative control plus three standard deviations. Only signals exceeding this threshold were considered above technical noise. In addition to the percentage of positive events, MFI values were compared between subjects without cardiovascular events and survivors of AMI and/or stroke. Relative expression was reported as fold change using the non-event group as the reference category (relative value = 1).

During assay optimization, Annexin V exhibited substantial nonspecific binding to capture beads in the absence of extracellular vesicles. Consequently, phosphatidylserine-positive vesicle analyses were excluded from the final study.

### 4.3. RNA Extraction from Plasma-Derived Small Extracellular Vesicles

Total RNA, including small RNA species, was isolated from plasma-derived extracellular vesicles using the exoRNeasy Maxi Kit (Cat. No. 77144, Qiagen, Hilden, Germany) according to the manufacturer’s protocol. Briefly, plasma samples were previously processed to remove cellular debris and larger extracellular vesicles as described above. One milliliter of plasma was mixed with an equal volume of Buffer XBP and loaded onto exoEasy spin columns. Following centrifugation at 500× *g* for 1 min at 4 °C, the flow-through was discarded and the membrane was washed with 10 mL of Buffer XWP.

Extracellular vesicles retained on the membrane were lysed directly by adding 700 μL of QIAzol reagent, followed by centrifugation at 5000× *g* for 5 min. The lysate was collected and mixed with 90 μL chloroform. After incubation at room temperature for 3 min, samples were centrifuged at 12,000× *g* for 15 min at 4 °C to achieve phase separation. The aqueous phase was transferred to a new tube, mixed with absolute ethanol, and loaded onto the purification column. Following several washing steps provided in the kit protocol, RNA was eluted in 12 μL of RNase-free water.

RNA concentration and purity were determined using a NanoDrop 2000 spectrophotometer (Thermo Fisher Scientific, Waltham, MA, USA). For samples subjected to next-generation sequencing, RNA integrity and size distribution were additionally evaluated using polyacrylamide gel electrophoresis and fluorometric quantification.

### 4.4. miRNA Expression Profiling Using miScript miRNA PCR Arrays and Next-Generation Sequencing

To comprehensively characterize the extracellular vesicle-associated small RNA cargo, small RNA libraries were prepared from 35 ng of total RNA using the MGIEasy RNA Library Prep Kit (MGI Tech, Shenzhen, China) following the manufacturer’s recommendations.

Briefly, reverse transcription generated double-stranded cDNA from small RNA molecules using the universal stem-loop reverse primer. Library preparation included end-repair, adapter ligation, amplification, and purification steps. Size selection was performed using magnetic bead purification to enrich fragments ranging from approximately 90 to 150 bp, corresponding to mature miRNAs and adapter sequences. Libraries were amplified by PCR (10–15 cycles), and their quality, size distribution, and concentration were assessed by polyacrylamide gel electrophoresis and fluorometric quantification.

For multiplex sequencing, unique molecular barcodes were incorporated into each library using the MGIEasy DNA Adapters-96 Kit (MGI Tech Co., Ltd., Shenzhen, China). Libraries meeting quality control criteria were circularized using the DNBLib Library Circularization Kit to generate DNA nanoballs (DNBs).

Sequencing was performed on the DNBSEQ-G50 platform using DNBSEQ-G50RS Sequencing Flow Cells and the DNBSEQ-G50RS High-Throughput Sequencing Kit (FCL SE100) (all from MGI Tech Co., Ltd., Shenzhen, China), generating single-end reads of 100 bp.

Raw sequencing data underwent quality control, adapter trimming, filtering of low-quality reads, and removal of contaminating sequences before downstream analyses. Demultiplexing was performed based on sample-specific barcode sequences incorporated during library preparation. Clean reads were aligned against reference human miRNA databases.

In addition, profiling of extracellular vesicle-associated miRNAs was performed using the miScript PCR System (Qiagen, GmbH, Hilden, Germany). For these trials, RNA pools were used at the same concentration for each of the four individuals per clinical group. Reverse transcription was carried out using 10 ng of total RNA and the miScript II RT Kit (Qiagen GmbH, Hilden, Germany) according to the manufacturer’s instructions. The resulting cDNA was diluted and used as template for amplification with miScript miRNA PCR Arrays (miRNOME Panels; Qiagen GmbH, Hilden, Germany), enabling simultaneous evaluation of a broad spectrum of human miRNAs.

Amplification reactions were performed following the manufacturer’s cycling recommendations. Amplification curves and melting profiles were examined to verify assay specificity and reaction performance. Relative miRNA expression levels were calculated using the comparative cycle threshold (2^−ΔCt^) method after normalization against the internal reference controls included in the array platform. Differentially expressed miRNAs identified during the screening phase were subsequently selected for independent validation.

### 4.5. Validation of Candidate miRNAs by Quantitative Real-Time PCR

Candidate miRNAs identified by array profiling and sequencing analyses were independently validated by quantitative real-time PCR (qPCR). Enriched small RNA fractions were subjected to poly(A) tailing and reverse transcription using a stem-loop poly(T)-based strategy. cDNA synthesis was performed using the GoScript™ Reverse Transcription System (Promega, Madison, WI, USA) and a universal stem-loop poly(T) primer designed to selectively amplify mature miRNAs.

Quantitative PCR reactions were performed using GoTaq^®^ qPCR Master Mix (Promega, Madison, WI, USA) in a final reaction volume of 20 μL containing miRNA-specific forward primers and a universal reverse primer complementary to the stem-loop sequence. Thermal cycling conditions consisted of an initial denaturation step at 95 °C for 2 min, followed by 40 amplification cycles of 95 °C for 15 s and 60 °C for 60 s.

The expression of the following miRNAs was evaluated: hsa-miR-126-3p, hsa-miR-126-5p, hsa-miR-16-5p, hsa-miR-21-5p, hsa-miR-29a-3p, hsa-miR-34a-5p, hsa-miR-99b-5p, hsa-miR-122-5p, hsa-miR-142-5p, hsa-miR-146a-5p, hsa-miR-155-5p, hsa-miR-208a-3p, hsa-miR-484, and hsa-miR-92a-3p. Primer sequences are listed in Table 3. and annotated miRNAs were quantified. Differential expression analyses were subsequently performed to identify extracellular vesicle-associated miRNAs linked to cardiometabolic phenotypes. Functional enrichment analyses of significantly deregulated miRNAs were conducted using established target prediction and pathway analysis tools to identify biological processes potentially involved in endothelial dysfunction, inflammation, atherosclerosis, and cardio-metabolic disease.

Relative expression levels were calculated using the comparative Ct method. For individual-sample analyses, expression values were calculated as 2^−ΔCt^ relative to the selected endogenous reference miRNA. miR-16-5p was used as the endogenous reference for normalization because of its stable expression across clinical groups.

### 4.6. Bioinformatic Analysis of Extracellular Vesicle-Associated miRNAs

Differentially expressed miRNAs identified by small RNA sequencing and subsequently validated by stem-loop RT-qPCR were subjected to bioinformatic analyses to investigate their potential biological functions and associated signaling pathways. Emphasis was placed on genes associated with endothelial dysfunction, vascular remodeling, inflammatory signaling, and EMT/EndMT pathways. Network analyses were therefore focused on target genes regulated by miR-126-3p, miR-21-5p, miR-92a-3p, and SNAIL-associated signaling pathways because these molecules exhibited the strongest differential expression patterns in extracellular vesicles derived from patients with cardiometabolic diseases.

#### 4.6.1. miRNA Target Gene Identification

Experimentally validated and predicted target genes of the selected miRNAs were identified using the online platform miRNet 2.0 (https://www.mirnet.ca 14 June 2026), an integrated network-based tool that combines information from multiple curated databases, including miRTarBase, miRecords, and miRanda. The list of significantly enriched miRNAs was uploaded into miRNet, and target gene retrieval was performed using the human genome reference (*Homo sapiens*). Both experimentally validated and high-confidence predicted interactions were included to maximize biological coverage while maintaining functional relevance.

#### 4.6.2. KEGG Pathway Enrichment Analysis and Network Analysis

Differentially expressed extracellular vesicle (EV)-associated miRNAs identified by small RNA sequencing and RT-qPCR validation were subjected to bioinformatic analyses to investigate their potential biological functions and regulatory networks. Predicted and experimentally validated miRNA–mRNA interactions were retrieved using miRNet 2.0, which integrates information from multiple curated databases, including miRTarBase, TarBase, and miRecords. Functional enrichment analysis was performed against the Kyoto Encyclopedia of Genes and Genomes (KEGG) database using a hypergeometric test followed by Benjamini–Hochberg false discovery rate (FDR) correction for multiple comparisons. Pathways with adjusted FDR values < 0.05 were considered significantly enriched. Enrichment results were visualized as bubble plots, in which pathway significance was represented by −log10(FDR), bubble size corresponded to the number of associated target genes, and color intensity reflected adjusted FDR values.

To further explore the regulatory landscape associated with the identified vesicular miRNA signature, miRNA–gene interaction networks were generated in miRNet using both predicted and experimentally validated target genes. Network analyses were used to identify shared molecular targets and biological pathways potentially involved in hypertension, type 2 diabetes mellitus, obesity, endothelial dysfunction, and cardiovascular disease. Gene Ontology (GO) Biological Process enrichment analysis was subsequently performed to identify overrepresented biological functions among the target genes. Enriched processes associated with inflammation, angiogenesis, endothelial dysfunction, extracellular matrix remodeling, fibrosis, epithelial-to-mesenchymal transition (EMT), endothelial-to-mesenchymal transition (EndMT), immune regulation, cell migration, apoptosis, and vascular homeostasis were interpreted in the context of cardiometabolic disease progression. All graphical outputs were generated using miRNet 2.0 and subsequently refined and formatted for publication using Adobe Illustrator (Adobe Inc., San Jose, CA, USA).

### 4.7. Translational Integration Framework

To provide a translational interpretation of the findings, epidemiological, anthropometric, metabolic, extracellular vesicle (EV), and bioinformatic data generated throughout the study were integrated into a unified analytical framework. Anthropometric, clinical, and biochemical variables were obtained from the cross-sectional cohort of 602 adults described above. Waist-to-height ratio (WHtR) was used as an indicator of central adiposity, and the triglyceride–glucose (TyG) index was calculated as a surrogate marker of insulin resistance.

The molecular component of the framework incorporated the characterization of plasma-derived EVs, including particle quantification, protein cargo analysis, surface marker evaluation (CD31), and EV-associated miRNA profiling. Differentially expressed miRNAs identified by small RNA sequencing and validated by stem-loop RT-qPCR were further subjected to target gene, pathway enrichment, and protein–protein interaction analyses. The methodologies used for epidemiological analyses, EV isolation and characterization, flow cytometry, miRNA profiling, and bioinformatic analyses are described in the corresponding sections above.

The purpose of this integrative approach was not to generate a single predictive model using all datasets simultaneously, but rather to examine the biological convergence between established cardiometabolic risk factors (WHtR and TyG), endothelial dysfunction (CD31-positive EVs), epithelial/endothelial-to-mesenchymal transition-related signaling (SNAIL), and EV-associated miRNAs. This translational framework was used to identify candidate biomarkers and molecular pathways potentially involved in the progression of cardio–metabolic disease.

### 4.8. Statistical Analysis

Due to the exploratory nature of molecular validation, sample sizes (per group) were chosen based on material availability and the goal of generating preliminary hypotheses. Consequently, *p*-values should be interpreted with caution.

Categorical variables were summarized as frequencies and percentages, whereas continuous variables were expressed as mean ± standard deviation (SD) or median with interquartile range (IQR), as appropriate. The normality of data distributions was assessed using the Shapiro–Wilk test.

For the epidemiological cohort (n = 602), comparisons between groups were performed using Student’s *t*-test or one-way analysis of variance (ANOVA) for normally distributed variables and Mann–Whitney U or Kruskal–Wallis tests for non-normally distributed variables. Associations between anthropometric, clinical, biochemical, and cardiometabolic variables were evaluated using Spearman’s or Pearson’s correlation coefficients, according to data distribution. Independent predictors of cardiometabolic risk were assessed using multivariable logistic regression models adjusted for relevant covariates, including age and sex. Odds ratios (ORs) and 95% confidence intervals (95% CIs) were calculated.

For extracellular vesicle (EV) characterization analyses, including nanoparticle tracking analysis (NTA), protein quantification, Western blot densitometry, and flow cytometry, comparisons among clinical groups were performed using one-way ANOVA followed by Tukey’s post hoc test when assumptions of normality were met, or Kruskal–Wallis tests followed by Dunn’s multiple-comparison correction for non-parametric data. Comparisons between subjects without previous cardiovascular events and survivors of acute myocardial infarction and/or ischemic stroke were performed using the Mann–Whitney U test. Paired analyses, when applicable, were conducted using the Wilcoxon signed-rank test. Associations between categorical variables were evaluated using Pearson’s chi-square or Fisher’s exact tests.

Relative miRNA expression levels obtained by stem-loop RT-qPCR were calculated using the 2^−ΔCt^ method and are presented as fold change relative to the control group. Because several molecular analyses were performed using pooled plasma samples, results should be interpreted as exploration and hypothesis-generating. Comparisons of relative miRNA expressions among cardiometabolic groups were performed using one-way ANOVA followed by Tukey’s multiple-comparison test.

For the exploratory endothelial marker analysis, fluorescence intensity values obtained by flow cytometry were normalized to the corresponding control group and expressed as fold change. Marker positivity thresholds were established using the mean signal of the negative control plus three standard deviations. Only signals exceeding this threshold were considered positive.

All statistical tests were two-tailed, and *p*-values < 0.05 were considered statistically significant. Statistical analyses were performed using GraphPad Prism version 10 (GraphPad Software, San Diego, CA, USA), R statistical software 3.6.3 version (R Foundation for Statistical Computing, Vienna, Austria), and IBM SPSS Statistics 27.0.1 version (IBM Corp., Armonk, NY, USA). Bioinformatic enrichment analyses employed Benjamini–Hochberg false discovery rate (FDR) correction, and pathways or Gene Ontology terms with adjusted FDR values < 0.05 were considered significantly enriched.

## 5. Conclusions

This study suggests that cardiometabolic disorders are associated with significant remodeling of circulating plasma-derived small extracellular vesicles, characterized by increased vesicle abundance, altered protein composition, enrichment of endothelial-associated markers, and selective loading of regulatory miRNAs involved in vascular homeostasis and inflammation. Hypertension emerged as the condition most strongly associated with EVs release, supporting the central role of endothelial dysfunction in vesicle biology.

The combined enrichment of miR-126-3p, miR-21-5p, miR-92a-3p, SNAIL, and CD31-positive vesicles identifies a molecular signature linked to endothelial injury, vascular remodeling, and residual cardiovascular risk. These findings suggest that circulating EVs function could be not only as biomarkers but also as potential mediators of intercellular communication during cardio-metabolic disease progression.

Overall, the integration of vesicle quantification, endothelial marker profiling, miRNA cargo characterization, and pathway enrichment analyses supports the potential utility of plasma-derived extracellular vesicles as liquid-biopsy platforms for early detection, risk stratification, and monitoring of cardiometabolic and cardiovascular diseases. Future large-scale and longitudinal studies are warranted to validate these candidates and establish their clinical applicability. These findings identify a molecular signature that requires independent validation in larger prospective cohorts before any claims of clinical utility can be made.

## Figures and Tables

**Table 1:** Characteristics of study subjects.

Characteristics	Main Findings	Epidemiological Interpretation
Subjects	602 adults from Riohacha, Maicao, and Manaure (La Guajira, Colombia); mean age: 58.6 ± 13.1 years; 65% women	Population characterized by social vulnerability and high cardiometabolic burden
Central adiposity (WHtR)	Mean Waist-to-Height Ratio (WHtR): 0.60 ± 0.08	Indicates high prevalence of abdominal obesity and visceral adiposity
Metabolic profile	Mean triglycerides: 139.7 ± 91.5 mg/dL; HbA1c: 6.22 ± 1.29%; homocysteine: 15.35 ± 8.27 µmol/L	Suggests frequent subclinical metabolic dysfunction
WHtR correlations	Positive correlation with triglycerides and fasting glucose; negative correlation with HDL cholesterol	Higher abdominal adiposity associated with an atherogenic metabolic profile
TyG index association	Positive correlation between WHtR and TyG index (rho = 0.0968; *p* = 0.0177)	Limited support for the link between visceral adiposity and insulin resistance
Hypertriglyceridemia	Individuals with elevated WHtR had higher prevalence of hypertriglyceridemia (33.8% vs. 11.9%)	Elevated triglycerides strongly associated with cardiometabolic risk
Hypertension	Hypertension was more frequent among participants with high WHtR	Reinforces the association between abdominal obesity and vascular dysfunction
Multivariable analysis	TyG index was the strongest independent predictor of elevated WHtR (adjusted OR = 2.76; 95% CI: 1.49–5.12)	TyG may identify early insulin resistance beyond clinically established diabetes
Public health implication	WHtR and TyG are low-cost, scalable, and easy-to-implement screening tools	Useful for early cardiometabolic risk detection in vulnerable and low-resource populations

Table 1. Resume of main characteristics of subjects studied, including cardiometabolic risks, anthropometric, and epidemiological interpretation. Main variables includedbody mass index (BMI), waist-to-height ratio (WHtR), lipid profile, HbA1c, triglyceride–glucose (TyG) index, and homocysteine levels. Cardiovascular events (myocardial infarction and stroke) were recorded. Data are presented as mean ± standard deviation or frequency (%). WHtR: waist-to-height ratio; TyG: triglyceride-glucose index; HbA1c: glycated hemoglobin.

**Table 3:** List of primers used in this study. The table reports the sequences of the primers employed for miRNA analysis, including poli T for RT reactions, forward for each miRNA evaluated and reverse primers used in qPCR assays.

miRNA	Sequence
hsa-miR-126-5p-F	5′ GGGGGGGCATTATTACTTTTG 3′
hsa-miR-208a-3p F	5′ CGC CCG CAT AAG ACG AGC AAA AA 3′
hsa-miR-126-3p F	5′ GGCGGCTCGTACCGTGAGTAAT 3′
hsa-miR-34a-5p F	5′CGGCTGGCAGTGTCTTAGCT 3′
hsa-miR-99b-5p F	5′ CGCCACCCGTAGAACCGAC 3′
hsa-miR-155-5p-F	5′ CGCCGTTAATGCTAATCGTG 3′
hsa-mir-16-5p	5′GGGAGTAGCAGCACGTAAA 3′
hsa-mir-21-5p	5′ GCGGGTAGCTTATCAGACT 3′
hsa-mir-92a-3p	5′ GGGGATTATTGCACTTGTCC 3′
hsa-mir-484	5′ CCGTCAGGCTCAGTCC 3′
hsa-mir-16-5p F	5′GGGAGTAGCAGCACGTAAA 3′
hsa-mir-21-5p F	5′ GCGGGTAGCTTATCAGACT 3′
hsa-mir-92a-3p F	5′ GGGGATTATTGCACTTGTCC 3′
hsa-mir-484 F	5′ CCGTCAGGCTCAGTCC 3′
hsa-miR-155-5p F	5′ CGC CTT AAT GCT AAT CGT GA 3′
hsa-miR-146a-5p F	5′ GGG GGC TGA GAA CTG AAT 3′
hsa-miR-29a-3p F	5′ CCC ACT AGC ACC ATC TGA A 3′
hsa-miR-27a-3p F	5′ GGG AAC TTC ACA GTG GCT A 3′
hsa-miR-122-5p F	5′ CTA GCG TGG AGT GTG ACA A 3′
hsa-miR-142-5p F	5′ GGG GGC ATA AAG TAG AAA G 3′
Stem-Loop poliT	5′GTCGTATCCAGTGCAGGGTCCGAGGTATTCGCACTGGATACGACTTTTTTTTTTTTVN 3′
Universal reverse	5′ CCA GTG CAG GGT CCG AGG TA 3′

## Data Availability

The original contributions presented in this study are included in the article/Supplementary Material. Further inquiries can be directed to the corresponding author.

## Supplementary Materials

The following supporting information can be downloaded at: https://www.mdpi.com/article/10.3390/ijms27156966/s1.
