# Supplementary material for: Integrated Epidemiological and Extracellular Vesicle Profiling Identifies miR-126-3p, miR-21-5p, miR-92a-3p and SNAIL as Candidate Biomarkers of Endothelial Dysfunction and Cardio-Metabolic Risk
Source: Int J Mol Sci. 2026 Aug 3;27(15):6966. doi: 10.3390/ijms27156966 (PMC13467177; doi:10.3390/ijms27156966)
Supplement: Supplementary file 1 [file ijms-27-06966-s001.zip › Supplementary Figure S1.pdf]

Supplementary Figure S1. Inclusion and exclusion criteria for individuals for molecular analysis

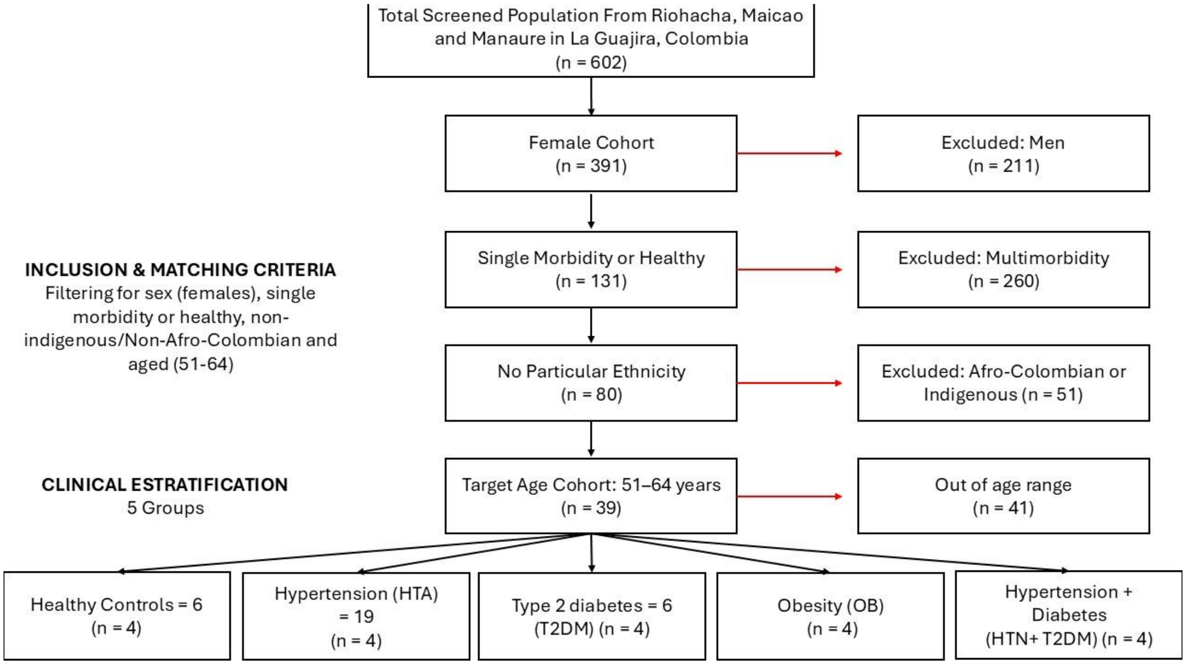

Figure S1. Selection criteria for the molecular analysis sub-cohort. For the molecular evaluations, a matched sub-cohort was selected to minimize biological heterogeneity. Inclusion criteria were: gender, age, clinical status, and ethnicity.

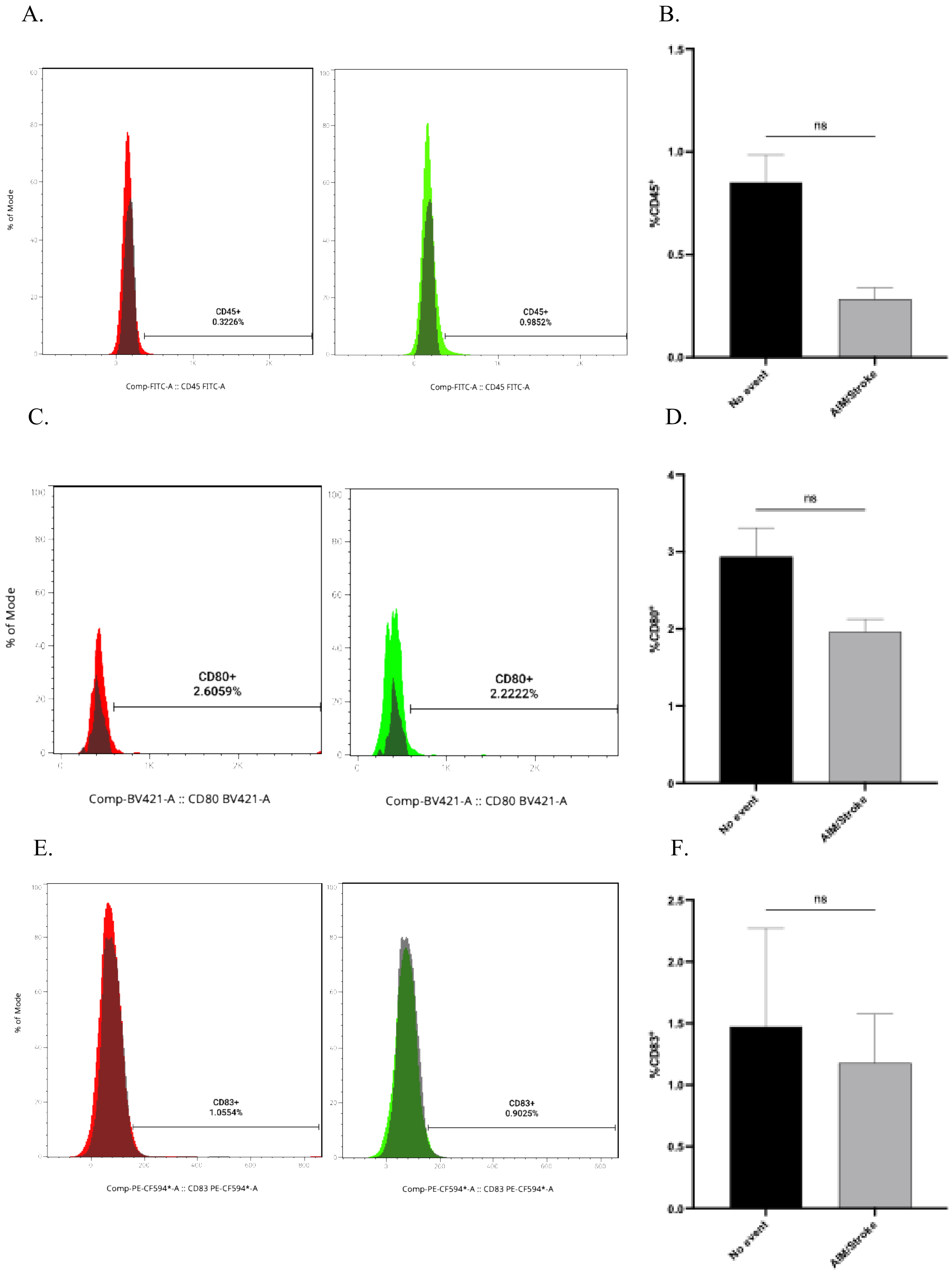
A.
B.
% of Mode
CD45+
0.3226%
Comp-FITC-A :: CD45 FITC-A
CD45+
0.9852%
Comp-FITC-A :: CD45 FITC-A
%CD45+
ns
No event
AMI/Stroke
C.
D.
% of Mode
CD80+
2.6059%
Comp-BV421-A :: CD80 BV421-A
CD80+
2.2222%
Comp-BV421-A :: CD80 BV421-A
%CD80+
ns
No event
AMI/Stroke
E.
F.
% of Mode
CD83+
1.0554%
Comp-PE-CF594*-A :: CD83 PE-CF594*-A
CD83+
0.9025%
Comp-PE-CF594*-A :: CD83 PE-CF594*-A
%CD83+
ns
No event
AMI/Stroke

Supplementary Figure S2. Representative CD45-FITC histograms and percentage of positivity of CD45 between groups (**A-B**). Representative CD80-BV421 histograms and percentage of positivity of CD80 between groups (**C-D**). Representative CD83-FITC histograms and percentage of positivity of CD83 between groups (**E-F**).
