# Supplementary material for: Integrated Epidemiological and Extracellular Vesicle Profiling Identifies miR-126-3p, miR-21-5p, miR-92a-3p and SNAIL as Candidate Biomarkers of Endothelial Dysfunction and Cardio-Metabolic Risk
Source: Int J Mol Sci. 2026 Aug 3;27(15):6966. doi: 10.3390/ijms27156966 (PMC13467177; doi:10.3390/ijms27156966)
Supplement: Supplementary file 1 [file ijms-27-06966-s001.zip › Supplementary tables.pdf]

Supplementary Table S1. Conceptual Framework of the Cardiometabolic Risk Continuum

Pathophysiological Domain	Evaluated Indicators	Underlying Biological Mechanism	Clinical Implications
Central adiposity	Waist-to-height ratio (WHtR)	Visceral fat accumulation associated with low-grade systemic inflammation and metabolic dysfunction.	Increased risk of insulin resistance, type 2 diabetes, and cardiovascular disease.
Insulin resistance	TyG index	Alterations in insulin signaling and glucose-lipid metabolism.	Higher probability of metabolic syndrome and cardiovascular disease.
General adiposity	Body mass index (BMI)	Positive energy balance and adipose tissue accumulation.	Significant risk factor for metabolic and cardiovascular diseases.
Glycemic homeostasis	Fasting glucose, HbA1c	Impaired glucose metabolism and endocrine control regulation.	Prediabetes and type 2 diabetes mellitus.
Lipid profile	Triglycerides, HDL-C, LDL-C, total cholesterol	Atherogenic dyslipidemia and impaired lipid transport.	Atherosclerosis, coronary artery disease, and global cardiovascular risk.

Table S1: This conceptual framework integrates anthropometric and biochemical indicators that reflect distinct components of the **pathophysiological continuum of the cardiometabolic syndrome,** allowing a comprehensive assessment of metabolic and cardiovascular risk.

Supplementary Table S2. Summary of Cardiometabolic Risk Indicators and Clinical Interpretation

Indicator	Pathophysiological Domain	Clinical Interpretation	Relevance for Cardiometabolic Risk Assessment
Waist-to-height ratio (WHtR)	Central adiposity	Elevated values indicate visceral fat accumulation.	Early predictor of cardiometabolic risk and cardiovascular disease.
TyG index	Insulin resistance	High values reflect alterations in glucose-lipid metabolism.	Surrogate marker of insulin resistance and predictor of type 2 diabetes.
Body mass index (BMI)	General adiposity	Allows classification of nutritional status (normal weight, overweight, obesity).	Widely used risk factor for metabolic diseases.
Fasting plasma glucose	Glycemic regulation	Elevated values indicate impaired glucose homeostasis.	Early indicator of prediabetes or diabetes.
HbA1c	Chronic glycemic exposure	Reflects the average glucose concentration over the past 2–3 months.	Key tool for diabetes diagnosis and monitoring.
Triglycerides	Lipid metabolism	Elevated levels indicate atherogenic dyslipidemia.	Associated with metabolic syndrome and cardiovascular risk.
HDL-C	Cardioprotective lipoprotein	Low values reflect decreased	Important factor in cardiovascular risk assessment.

Indicator	Pathophysiological Domain	Clinical Interpretation	Relevance for Cardiometabolic Risk Assessment
		vascular protection.	
LDL-C	Atherogenic lipoprotein	Elevated concentrations promote atherosclerotic plaque formation.	Primary therapeutic target in cardiovascular prevention.
Total cholesterol	Global lipid load	Elevated values reflect alterations in lipid metabolism.	Overall indicator of cardiovascular disease risk.

Table S2: The clinical interpretation of these indicators is based on widely accepted international guidelines for cardiometabolic risk assessment, including recommendations from the World Health Organization (WHO), the American Diabetes Association (ADA), and the National Cholesterol Education Program (NCEP-ATP III).

Supplementary Table S3. Conceptual Framework of the Cardiovascular-Metabolic (CM) Syndrome

Pathophysiological Domain	Clinical Components	Main Biological Mechanisms	Clinical Consequences
**Adiposity and metabolic dysfunction**	Obesity, visceral adiposity, metabolic syndrome.	Chronic low-grade inflammation, lipotoxicity, and adipose tissue endocrine alterations.	Increased risk of type 2 diabetes and cardiovascular disease.
**Glycemic dysregulation**	Insulin resistance, hyperglycemia, diabetes.	Alterations in insulin signaling and oxidative stress.	Vascular damage and progression of cardiovascular disease.
**Atherogenic dyslipidemia**	Elevated triglycerides, high LDL-C, low HDL-C.	Alterations in lipid metabolism and lipoprotein transport.	Atherosclerosis and coronary artery disease.
**Cardiovascular dysfunction**	Hypertension, atherosclerosis, structural heart disease.	Vascular remodeling, oxidative stress, and endothelial dysfunction.	Heart failure and major adverse cardiovascular events (MACE).

Table S3: Cardiovascular-Metabolic (CM) syndrome represents an integrative conceptual framework that recognizes the bidirectional interplay between metabolic dysfunction and cardiovascular disease, highlighting the need for integrated preventive and therapeutic approaches in public health and clinical medicine, in accordance with the recent scientific statement from the American Heart Association (AHA).

Supplementary Table S4. Integrated clinical, anthropometric, metabolic, and extracellular vesicle-associated molecular predictors of cardiometabolic risk.

Variable	Category	Adjusted OR	95% CI	p-value	Main Biological Interpretation
TyG index	Metabolic	2.76	1.49–5.12	0.001	Marker of insulin resistance and metabolic dysfunction
WHtR	Anthropometric	2.10	1.50–2.95	<0.001	Indicator of visceral adiposity
Hypertension	Clinical	2.30	1.05–5.04	0.038	Associated with vascular dysfunction
miR-126-3p	EV-associated miRNA	1.85	1.30–2.60	<0.001	Endothelial homeostasis and vascular repair
miR-92a-3p	EV-associated miRNA	1.72	1.20–2.46	0.004	Endothelial dysfunction and vascular remodeling
miR-21-5p	EV-associated miRNA	1.58	1.11–2.25	0.012	Inflammation, fibrosis, and tissue remodeling

Table S4: Multivariable logistic regression models integrating anthropometric, metabolic, clinical, and extracellular vesicle-associated molecular variables. Odds ratios (ORs) were adjusted for age, sex, waist-to-height ratio (WHtR), and triglyceride–glucose (TyG) index. EVs-associated miRNA estimates should be interpreted as exploratory due to pooled-sample analysis. TyG: triglyceride–glucose index; WHtR: waist-to-height ratio; EVs: extracellular vesicles.

Supplementary Table S5. Translational integration of extracellular vesicle biomarkers with cardiometabolic and cardiovascular phenotypes.

Biomarker	EV-associated Finding	Clinical Association	Potential Pathophysiological Process
CD31 (PECAM-1)	Increased fluorescence trend in AMI/Stroke survivors ($p = 0.057$)	Previous cardiovascular events	Endothelial activation and vascular injury
SNAIL	Increased vesicular expression in HTA and OB	Cardiometabolic disease	EMT/EndMT and tissue remodeling
miR-126-3p	Progressive increase across disease spectrum	HTA, HTA+DM	Endothelial dysfunction and vascular homeostasis
miR-92a-3p	Markedly elevated in hypertensive groups	HTA, HTA+DM	Vascular remodeling and endothelial stress
miR-21-5p	Strong increase in cardiometabolic disease	HTA, HTA+DM	Inflammation, fibrosis, and remodeling
Increased EV abundance	Highest concentrations in HTA	Cardiometabolic dysfunction	Enhanced vesicle release under vascular stress

Table S5: Summary of extracellular vesicle-associated candidate biomarkers identified throughout the study and their relationship with endothelial dysfunction, inflammation, epithelial/endothelial-to-mesenchymal transition (EMT/EndMT), and cardiovascular disease progression. Findings are derived from EVs characterization, miRNA profiling, protein cargo analyses, and exploratory flow cytometry experiments.

Supplementary Table S6. Conceptual translational framework linking epidemiological, metabolic, endothelial, and candidate's extracellular vesicle-associated biomarkers.

Biological Level	Principal Findings	Representative Biomarkers
Population/Epidemiological	High cardiometabolic burden	WHtR, TyG, triglycerides, hypertension
Metabolic Dysfunction	Insulin resistance and adiposity	TyG index, WHtR
Endothelial Dysfunction	Increased endothelial-derived EV signatures	CD31-positive EVs
Vesicular Cargo Remodeling	Altered EV molecular composition	miR-126-3p, miR-92a-3p, miR-21-5p
EMT/EndMT Signaling	Increased remodeling-associated proteins	SNAIL
Functional Pathways	PI3K-Akt, MAPK, NF-κB, VEGF, TGF-β, Rap1, JAK-STAT	KEGG and network analyses
Clinical Consequence	Cardiovascular and cardio-reno-metabolic disease progression	AMI, stroke, HTN, T2DM

Table S6: Integrated conceptual framework summarizing the convergence of epidemiological, anthropometric, metabolic, extracellular vesicle, endothelial, and bioinformatic findings. The table illustrates the proposed biological continuum linking visceral adiposity and insulin resistance with endothelial dysfunction, extracellular vesicle remodeling, inflammatory signaling, and progression toward cardio-reno-metabolic disease.
